# KMT2A and chronic inflammation as potential drivers of sporadic parathyroid adenoma

**DOI:** 10.1002/ctm2.1734

**Published:** 2024-06-18

**Authors:** Qin Xu, Ting La, Kaihong Ye, Li Wang, Shasha Wang, Yifeng Hu, Liu Teng, Lei Yan, Jinming Li, Zhenhua Zhang, Zehua Shao, Yuan Yuan Zhang, Xiao Hong Zhao, Yu Chen Feng, Lei Jin, Mark Baker, Rick F. Thorne, Xu Dong Zhang, Feng‐Min Shao, Huixia Cao

**Affiliations:** ^1^ Department of Nephrology, Henan Key Laboratory of Kidney Disease and Immunology of Zhengzhou University People's Hospital Zhengzhou University People's Hospital ,Henan Provincial People's Hospital Zhengzhou China; ^2^ National‐Local Joint Engineering Research Center of Biodiagnosis & Biotherapy The Second Affiliated Hospital Xi'an Jiaotong University Xi'an China; ^3^ Translational Research Institute Henan Provincial and Zhengzhou City Key Laboratory of Non‐Coding RNA and Cancer Metabolism Henan International Join Laboratory of Non‐Coding RNA and Metabolism in Cancer Zhengzhou University People's Hospital and Henan Provincial People's Hospital Academy of Medical Sciences Zhengzhou University Zhengzhou China; ^4^ School of Basic Medical Sciences Zhengzhou University Zhengzhou China; ^5^ Department of Nephrology Xinxiang Medical University Xinxiang China; ^6^ Department of Thyroid Surgery Henan Provincial People's Hospital Zhengzhou University People's Hospital Zhengzhou China; ^7^ Children's Heart Center Henan Provincial People's Hospital Zhengzhou University People's Hospital Zhengzhou China; ^8^ School of Biomedical Sciences and Pharmacy The University of Newcastle Callaghan New South Wales Australia; ^9^ School of Medicine and Public Health The University of Newcastle Callaghan New South Wales Australia

**Keywords:** CCND2, cyclin D2, epigenetics, KMT2A, single‐cell analysis, sporadic parathyroid adenoma

## Abstract

**Background:**

Sporadic parathyroid adenoma (PA) is the most common cause of hyperparathyroidism, yet the mechanisms involved in its pathogenesis remain incompletely understood.

**Methods:**

Surgically removed PA samples, along with normal parathyroid gland (PG) tissues that were incidentally dissected during total thyroidectomy, were analysed using single‐cell RNA‐sequencing with the 10× Genomics Chromium Droplet platform and Cell Ranger software. Gene set variation analysis was conducted to characterise hallmark pathway gene signatures, and single‐cell regulatory network inference and clustering were utilised to analyse transcription factor regulons. Immunohistochemistry and immunofluorescence were performed to validate cellular components of PA tissues. siRNA knockdown and gene overexpression, alongside quantitative polymerase chain reaction, Western blotting and cell proliferation assays, were conducted for functional investigations.

**Results:**

There was a pervasive increase in gene transcription in PA cells (PACs) compared with PG cells. This is associated with high expression of histone‐lysine N‐methyltransferase 2A (KMT2A). High KMT2A levels potentially contribute to promoting PAC proliferation through upregulation of the proto‐oncogene *CCND2*, which is mediated by the transcription factors signal transducer and activator of transcription 3 (STAT3) and GATA binding protein 3 (GATA3). PA tissues are heavily infiltrated with myeloid cells, while fibroblasts, endothelial cells and macrophages in PA tissues are commonly enriched with proinflammatory gene signatures relative to their counterparts in PG tissues.

**Conclusions:**

We revealed the previously underappreciated involvement of the KMT2A‒STAT3/GATA3‒*CCND2* axis and chronic inflammation in the pathogenesis of PA. These findings underscore the therapeutic promise of KMT2A inhibition and anti‐inflammatory strategies, highlighting the need for future investigations to translate these molecular insights into practical applications.

**Highlights:**

- Single‐cell RNA‐sequencing reveals a transcriptome catalogue comparing sporadic parathyroid adenomas (PAs) with normal parathyroid glands.
- PA cells show a pervasive increase in gene expression linked to KMT2A upregulation.
- KMT2A‐mediated STAT3 and GATA3 upregulation is key to promoting PA cell proliferation via cyclin D2.
- PAs exhibit a proinflammatory microenvironment, suggesting a potential role of chronic inflammation in PA pathogenesis.

## Graphical abstract

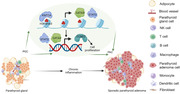

## 1 INTRODUCTION

The parathyroid glands (PGs) are essential for maintaining serum calcium concentrations within a narrow physiological range through secretion of parathyroid hormone (PTH).^1^ Circulating PTH activates osteoclasts, resulting in the resorption of bone to release calcium into the circulation.^1^ PTH also promotes calcium reabsorption and inhibits the reabsorption of phosphate in kidneys, leading to decreases in the plasma phosphate concentrations and thus increased amounts of ionised calcium.^1^ Moreover, PTH stimulates 25‐hydroxy vitamin D conversion into active 1,25‐dihydroxy vitamin D, which in turn enhances calcium uptake from the intestine.^1^ The production and secretion of PTH are tightly controlled by serum calcium ion concentrations through negative feedback mediated by calcium‐sensing receptor (CASR) expressed on PG cell (PGC) surface.^2^, ^3^

Primary hyperparathyroidism (pHPT) is a pathological condition in which one or more of the PGs secretes excessive amounts of PTH, leading to hypercalcaemia.^4^ Although pHPT can be caused by parathyroid hyperplasia and several hereditary endocrine disorders, including multiple endocrine neoplasia type 1 (MEN1) and type 2A (MEN2A), sporadic single parathyroid adenoma (PA) is by far the most common cause of the disease.^4^, ^5^, ^6^ Parathyroidectomy remains the most effective approach in PA treatment, whereas calcimimetics that allosterically activate CASR to reduce PTH secretion are recommended for patients who are contraindicated for surgery.^7^, ^8^

The pathogenesis of PA is not completely understood but can arise from either chief or oxyphil cells, representing different states of PTH‐producing PGCs.^1^, ^4^, ^9^, ^10^ In some PAs, translocation of the *CCND1* gene results in oncogenic upregulation of its protein product cyclin D1 that is essential for G1/S cell cycle transition,^6^, ^8^, ^9^ while the mutational loss of tumour suppressor genes has also been found, including the genes *MEN1*, *tumour protein 53* (*TP53*) and *cyclin‐dependent kinase inhibitor 1B* (*CDKN1B*).^11^, ^12^, ^13^, ^14^ Moreover, epigenetic mechanisms have been identified to contribute to PA development and progression.^15^, ^16^ For example, DNA hypermethylation inactivates the tumour suppressor genes, *cyclin‐dependent kinase inhibitor 2A* (*CDKN2A*), *hypermethylated in cancer 1* (*HIC1*), *PR/SET domain 2* (*PRDM1*) and *Ras association domain family member 1* (*RASSF1*), whereas the increased expression of the histone‐lysine N‐methyltransferase, enhancer of zeste homologue 2 (EZH2), promotes Wnt/β‐catenin signalling and proliferation in PA cells (PACs).^17^, ^18^, ^19^, ^20^, ^21^ Of further note, immune cell infiltration has also been well documented in PA,^22^, ^23^ although its potential involvement or the involvement of other microenvironmental factors in the pathogenesis of PA remains to be clarified.

Single‐cell RNA‐sequencing (scRNA‐seq), a technology offering several advantages over traditional bulk RNA‐seq, such as high resolution and cellular heterogeneity insight, has been increasingly used to analyse the transcriptomic landscape of individual cells under various pathophysiological conditions.^24^, ^25^, ^26^, ^27^ In particular, its advantages for exploring the underlying mechanisms of PA pathogenesis have been demonstrated.^28^, ^29^ However, the absence of normal PGCs as a control in previous scRNA‐seq analyses of PACs has made it difficult to interpret the results in a comparative context.^28^, ^29^

We have carried out scRNA‐seq analysis to compare the cellular compositions and the molecular characteristics of individual cell types between PA and PG tissues. We show here that there is a widespread increase in gene transcription in PACs compared with PGCs. This is associated with high expression of the histone methyltransferase, histone‐lysine N‐methyltransferase 2A (KMT2A; also known as acute lymphoblastic leukaemia 1 or myeloid/lymphoid or mixed‐lineage leukaemia 1). High levels of KMT2A may play a critical part in promoting PAC proliferation through transcriptional upregulation of the proto‐oncogene *CCND2*, which is mediated by the transcription factors (TFs) signal transducer and activator of transcription 3 (STAT3) and GATA binding protein 3 (GATA3). Furthermore, we demonstrated that fibroblasts, endothelial cells (ECs), and macrophages in the PA microenvironment are enriched with proinflammatory gene signatures as opposed to their counterparts in PGs, highlighting the potential role of chronic inflammation in PA pathogenesis.

## 2 METHODS AND MATERIALS

### 2.1 Patient samples and cell lines

All sporadic PA patients were diagnosed on the basis of standard clinical and biochemical parameters, including serum calcium and PTH levels as well as ultrasonographic examination, in the absence of other possible causes of hypercalcaemia (Figure S1A). Pathological verification of the diagnosis was achieved through frozen section examination during operation. The relative oxyphil cell content was quantified by examining formalin‐fixed paraffin‐embedded (FFPE) tissue sections from each PA sample stained with haematoxylin and eosin (H&E) (Figure S1A). We did not identify any indicators of chronic inflammation, such as obesity or abnormal blood counts, in the patients included in this study. Similarly, histological examination of the H&E‐stained sections of the PA tissues revealed no unusual features. Normal PG tissues were incidentally obtained from total thyroidectomy surgical specimens from patients with pathologically diagnosed thyroid cancer. FFPE PA tissues were obtained from the Pathology Department of Henan Provincial People's Hospital. The human embryonic kidney epithelial‐like cell line HEK293T, cervical adenocarcinoma cell line HeLa and human thyroid follicular epithelial cell line Nthy‐ori 3‐1 were obtained from ATCC (CRL‐3216 and CCL‐2; RRID: CVCL_0063, CVCL_0030 and CVCL_2659, respectively) and maintained in Dulbecco'd modified eagle medium (DMEM) containing 10% fetal calf serum (FCS) (Thermo Fisher), 100 U/mL penicillin and 100 µg/mL streptomycin. The cell line was verified to be mycoplasma‐free using polymerase chain reaction (PCR) every 3 months and was validated using short tandem repeat profiling through Henan Engineering Research Center of Industrial Microbiology.

### 2.2 Primary PAC culture

Fresh PA tissues, obtained from the operating room, were immediately placed into a phosphate buffer saline (PBS) solution containing penicillin and streptomycin. These tissues were then dissociated, washed twice using PBS supplemented with penicillin and streptomycin and subsequently minced into very small pieces using sterile scissors. Following the washes, the minced PA tissue was digested with a collagenase II digestion solution and incubated in a 37°C incubator for 30 min, with agitation every 5 min. Afterward, 5 mL of trypsin digestion solution containing ethylene diamine tetraacetic acid (EDTA) was added, and the incubation continued at 37°C for an additional 15 min with vortexing every 5 min. Five millilitres of red blood cell lysis buffer was then added to suspend and lyse the red blood cells. The dissociated cells were maintained in DMEM‒F12 Ham's modified growth medium (Thermo Fisher) supplemented with 20 µg/mL bovine pituitary extract (Absin), 120 ng/mL epidermal growth factor (Peprotech), 3 µg/mL transferrin (Merck), 10 µg/mL insulin (Beyotime), 2.5 ng/mL hydrocortisone (MCE) and 1% penicillin‒streptomycin (Solarbio) at 37°C in a 5% CO_2_ environment.

### 2.3 Reagents and antibodies

Information on reagents and antibodies (Abs) used is supplied in Tables S1 and S2, respectively.

### 2.4 Single‐cell suspension preparation

Freshly removed samples were rinsed with PBS and transported in ice‐cold tissue storage solution (Miltenyi Biotec) to the laboratory. Every sample was then minced to less than 1 mm cubic pieces on ice. These pieces were digested using a mixture containing 2 mL of Gibco trypsin/EDTA solution consisting of .01% EDTA and .025% trypsin (Thermo Fisher) and 1 mL of collagenase II (1 mg/mL; Biofroxx). After centrifugation, the cells were resuspended in PBS without calcium and magnesium containing .04% bovine serum albumin (BSA) (Thermo Fisher). The cells were then resuspended in 1 mL of ice‐cold red blood cell lysis buffer (Miltenyi Biotec) followed by centrifugation at 300 *g* for 5 min, and then incubated for 10 min at 4°C. This was followed by the addition of 10 mL of ice‐cold PBS and centrifugation for 10 min at 300 *g*. After resuspension, each sample was filtered with a 40 µm cell strainer (Miltenyi Biotec). Cell viability was then determined using Trypan blue (15250061, Thermo Fisher) staining with an automated cell counter (Thermo Fisher).

### 2.5 Droplet‐based scRNA‐seq

The Chromium Next GEM Single cell 3′ Reagent v3.1 kit was employed for preparing barcoded scRNA‐seq libraries according to the manufacturer's protocol (10× Genomics). Single‐cell suspensions were added onto a Chromium Single‐Cell Controller Instrument (10× Genomics) to give rise to single‐cell gel beads in emulsions. Reverse transcription reactions were engaged to produce barcoded full‐length cDNA. This was followed by emulsion disruptions with the recovery agent. Clean‐up of cDNA was the performed with Myone Silane Beads (Thermo Fisher), followed by cDNA amplification through PCR. The cDNA was then fragmented, end‐repaired, A‐tailed and ligated to an index adaptor. The library was amplified for further sequencing emplying the NovaSeq platform (Illumina). A total of 150 bp paired‐end reads were generated for further analysis. The data produced by scRNA‐seq were deposited in the Gene Expression Omnibus under accession code GSE190773.

### 2.6 Processing and quality control of the raw data

Cell Ranger (version 5.0.0; 10× Genomics) was employed for processing the raw data, demultiplex cellular barcodes, map reads to the transcriptome and down‐sample reads (as required to generate normalised aggregate data across samples). The processes generated a raw UMI count matrix that was converted to a Seurat object by the R package Seurat (version 3.1.1). Cells with UMI numbers <500, over 50 000, or gene numbers <350, over 6500, or with over 25% UMI counts generated by mitochondrial transcripts were regarded as cells with low quality and were abandoned (Figure S1B). We also processed our scRNA‐seq data using the adaptively thresholded low‐rank approximation method, which decomposes the gene expression matrix into a lower‐dimensional representation while adaptively choosing a threshold for the singular values obtained during the decomposition, thus diminishing the influence of noise and dropouts while retaining the significant structure of the data.^30^

### 2.7 Quantification of single‐cell gene expression and cell type determination

The UMI count matrix was log normalised by the NormalizeData function following quality control. Patient ID was employed to eliminate batch effects. Then, the top 3000 genes were included to generate potential anchors with FindVariableFeatures function of Seurat. The Seurat object was then integrated using the CCA method and a new matrix with 3000 features was created by using the integrateData, where potential batch effects were regressed out. Principal components were calculated for the selected genes and then projected onto all other genes with the RunPCA commands. Graph‐based clustering was conducted for clustering cells based on the expression profile of genes using the FindClusters function of Seurat. The Run uniform mainfold approximation and projection (UMAP) function of Seurat was employed for reducing the dataset dimensionality. Two‐dimensional UMAP plots were generated by Dimplot. Violin plots were obtained employing the VlnPlot function of Seurat, and the width of data distribution band was assessed using kernel density estimation, as per the built‐in ‘nrd0’ option. Given the data size, individual data points were not displayed on violin or box plots to avoid obscuring the overall distribution.

### 2.8 Trajectory analysis

We used single‐cell trajectory analysis with the R package Monocle2 to explore the adenoma‐reprogramming processes of parathyroid. We used ‘newCellDataSet’ function to construct the monocle subject based on differentially expressed genes, applied the ‘reduceDimension’ function to reduce dimensions and taken ‘orderCells’ functions to place cells onto a pseudotime trajectory.

### 2.9 single‐cell regulatory network inference and clustering analysis

We conducted single‐cell regulatory network inference and clustering (SCENIC) analysis using pySCENIC (version 0.9.18). The correlation coefficients between regulators/TFs and their targets were calculated using the entire expression matrix to generate multiple modules and the positively correlated and most stable modules were retained for further analysis. The co‐expression modules were pruned by cis‐regulatory motif analysis using RcisTarget to exclude the indirect targets. Direct target genes across all modules sharing the same TF were combined into a single resulting regulon. The activity of the predicted regulon was quantified using the area under the recovery curve.

### 2.10 Gene set variation analysis

Gene set variation analysis (GSVA) of hallmark pathway gene signatures was carried out on the 50 hallmark pathways contained in the molecular signature database derived from the GSEABase package (version 1.36.0). Each pathway‐associated gene set was modified to encompass only unique genes. The genes associated with two or more pathways were eliminated from the analysis. A gene set that was related to a pathway was trimmed to encompass only unique genes. Genes related to two or more pathways were abandoned from the analysis. Estimates of pathway activity in individual cells were conducted with standard settings implemented in the GSVA package. *z*‐Scores were converted from GSVA scores.

### 2.11 Cell‐to‐cell communications

Interactions among cells according to the expression of established ligand–receptor pairs were inferred by employing CellChat (version 1.12).^31^ The normalised transcript counts were loaded into CellChat following the official workflow.^31^ The functions of identifyOverExpressedGenes, identifyOverExpressedInteractions and projectData were then utilised. The signalling pathways secreted and precompiled human protein–protein interactions were chosen as priori network information. The core functions of ComputeCommunProb, ComputeCommunProbPathway and aggregateNet were then performed with standard parameters and fixed randomisation seeds, which was followed by the application of the function netAnalysis_signallingRole to define the receivers and senders in the network.

### 2.12 Immunohistochemistry

Immunohistochemistry (IHC) was carried out as described previously.^32^ In brief, 5‐µm‐thick sections were cut from each FFPE tissue block, followed by deparaffinisation and rehydration. The retrieval of antigen was carried out using boiling sodium citrate buffer solution (.01 M, pH 6.0). Endogenous peroxidase activity was blocked using 3% H_2_O_2_ at room temperature, followed by washing in PBS. Nonspecific binding was blocked by incubation with a blocking buffer (5% BSA in PBS). The sections were then incubated with primary Abs overnight at 4°C, followed by washing with PBS and incubation with a biotinylated secondary Ab. The slides were then incubated with streptavidin‒peroxidase complex followed by diaminobenzidine (DAB). Counterstaining was carried out using hematoxylin. Cytoseal 60 (Thermo Fisher) was used to mount sections after dehydration. Examination of slides were performed by two independent investigators who were blinded to the sample grouping. The proportion of positive cells was assessed on an arbitrary scale of 0−4: no positive cells (0), <10% of positive cells (1), 10%−50% of positive cells (2), 51%‒80% of positive cells (3) and >80% of positive cells (4). The intensity of staining (intensity score) was recorded on an arbitrary scale of 0‒3: no staining (0), weakly positive staining (1), moderately positive staining (2) and strongly positive staining (3). An immunoreactive score (IRS) was obtained by multiplying the score of the percentage of positive cells by the staining intensity.

### 2.13 Immunofluorescence

Five‐micrometre‐thick FFPE tissue sections were deparaffinised and rehydrated, followed by antigen retrieval using boiling sodium citrate buffer solution (.01 M, pH 6.0). The sections were then incubated with primary Abs at 4°C overnight. After washing, sections were incubated with a fluorescence conjugated secondary Ab at room temperature in the dark for 60 min. The slides were mounted using the ProLongTM Glass Antifade Mountant with NucBlue reagent (Thermo Fisher). Images were recorded digitally by employing a Leica SP8 confocal microscope (Leica Camera AG).

### 2.14 siRNA

siRNAs were purchased from GenePharma. They were transfected by the use of the lipofectamine 3000 Transfection Kit (Thermo Fisher Scientific, #L3000‐075) as previously described.^33^ Information on KMT2A siRNA, GATA3 siRNA and STAT3 siRNA sequences is provided in Table S3.

### 2.15 Plasmids

The pcDNA3.1(+)‐KMT2A and pcDNA3.1(+)‐CCND2 plasmids were constructed by BGI.

### 2.16 Western blotting

The procedure has been described previously.^34^ In brief, cells were harvested and lysed with cell lysis buffer containing 10 mM Tris‒HCl (pH 7.6), 140 mM NaCl, .5 mM CaCl_2_, .5 mM MgCl_2_, .02% NaN_3_, 1% Triton X‐100 and a proteinase inhibitor cocktail (#12352200, Roche). Equal amounts of the protein were subjected to sodium dodecyl sulfate polyacrylamide gel electrophoresis (SDS‒PAGE followed by analysis with Western blotting.

### 2.17 Cell proliferation

Cell proliferation was quantitated by counting cell numbers using a hemocytometer (Thermo Fisher).

### 2.18 Statistics

Box plots were produced employing the R base package ggplot. The boxes span the interquartile range (IQR; from the 25th to the 75th percentiles), with the centerline conforming to the median. Lower whiskers stand for the data minimum or the 25th percentile minus 1.5 × IQR, whichever is greater. Upper whiskers symbolise the data maximum or the 75th percentile plus 1.5 × IQR (lower), whichever is lower. Heatmaps were created with the ComplexHeatmap package. All other analyses were carried out using the GraphPad Prism 9. Comparisons between two groups were carried out with unpaired two‐tailed *t*‐tests. One‐way analysis of variance with Tukey's multiple comparisons tests were employed for multiple group comparisons. *p*‐Values less than .05 were regarded as statistically significant.

## 3 RESULTS

### 3.1 Cell typing of PA relative to PG tissues through scRNA‐seq

**FIGURE 1:**
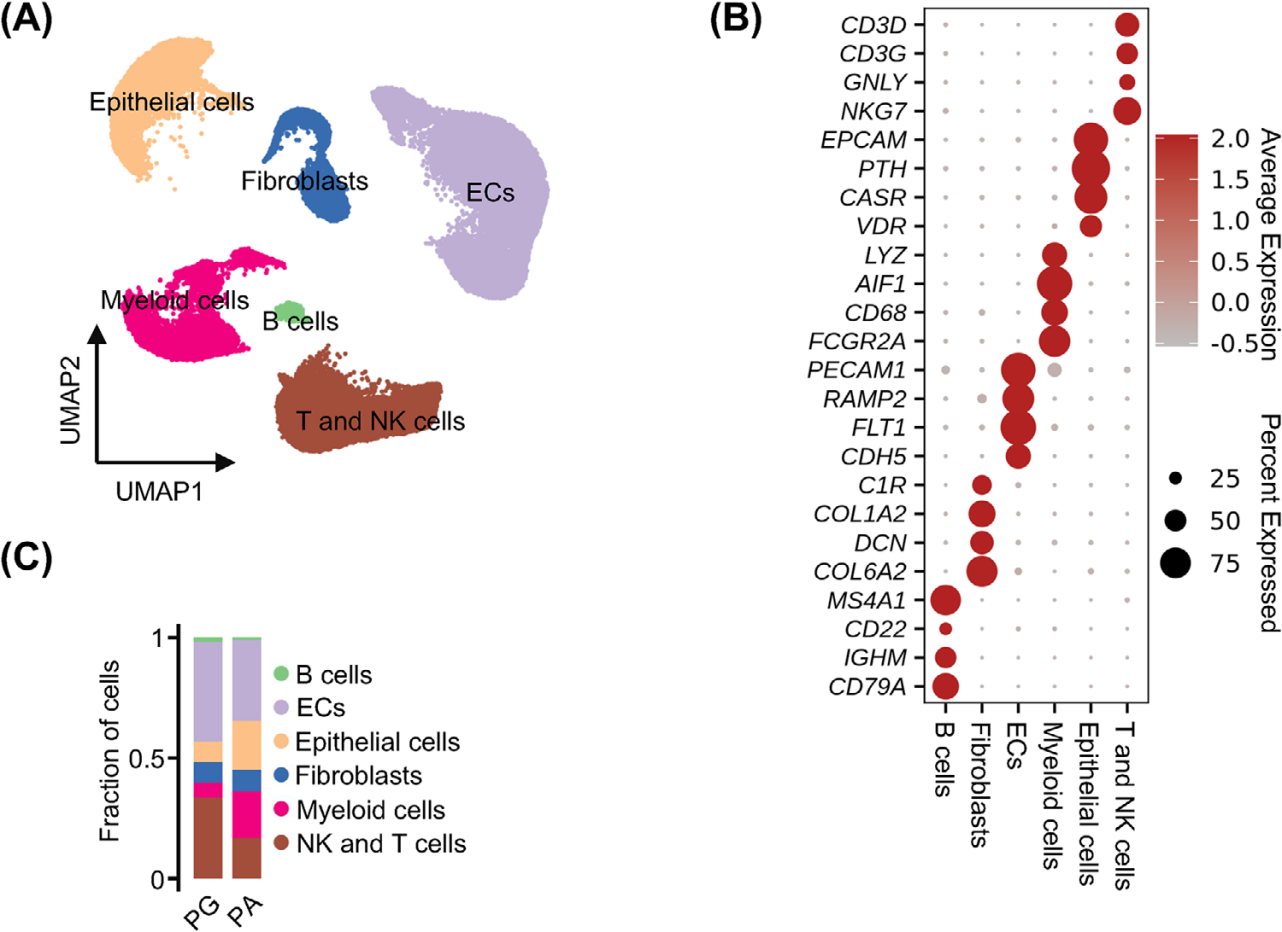
Cell typing of parathyroid adenoma (PA) relative to parathyroid gland tissues using single‐cell RNA‐sequencing (scRNA‐seq). (A) UMAP plots of the 49 819 cells profiled with each cell colour‐coded for cell types. (B) Dot plots of cell type marker genes. Expression values were normalised and scaled to the averages. (C) Proportion plots of major cell types in PG and PA tissues.

To characterise the cellular components of PA relative to PG tissues, we conducted scRNA‐seq analysis of surgically removed sporadic PA samples from five patients (patients 1−5) (Figure S1A) and normal PG tissues from three individuals who were incidentally dissected during total thyroidectomy. Using the 10× Genomics Chromium Droplet platform, we detected ∼.27 billion unique transcripts from 49 819 cells in which more than 300 genes were recorded (Figure S1B). Principal component analysis of differentially expressed genes (*n* = 1842 genes) classified the cells into 13 clusters that were readily allocated to known cell lineages through marker genes, including epithelial cells (PACs and PGCs; 8083 cells), ECs (18 076 cells), fibroblasts (4419 cells), myeloid cells (7227 cells), T lymphocytes (T cells) and natural killer cells (NK cells) (11 372 cells), with a relatively small number of B cells (642 cells) (Figures 1A‒C and S2A,B).

Although every cell type was represented in all samples, the frequency of each cell type varied widely across the PA and PG samples (Figures 1C and S2B). When compared collectively, the proportions of epithelial cells and myeloid cells markedly increased, whereas the fractions of ECs and T/NK cells decreased, in PA compared to PG samples (Figure 1C). The presence of representative cell types in PA tissues was visually confirmed using IHC in independent FFPE PA tissue sections (Figure S2C). Together, these results demonstrate the differences in the cellular compositions between PA and PG tissues.

### 3.2 A pervasive increase in transcriptional activity in PACs

**FIGURE 2:**
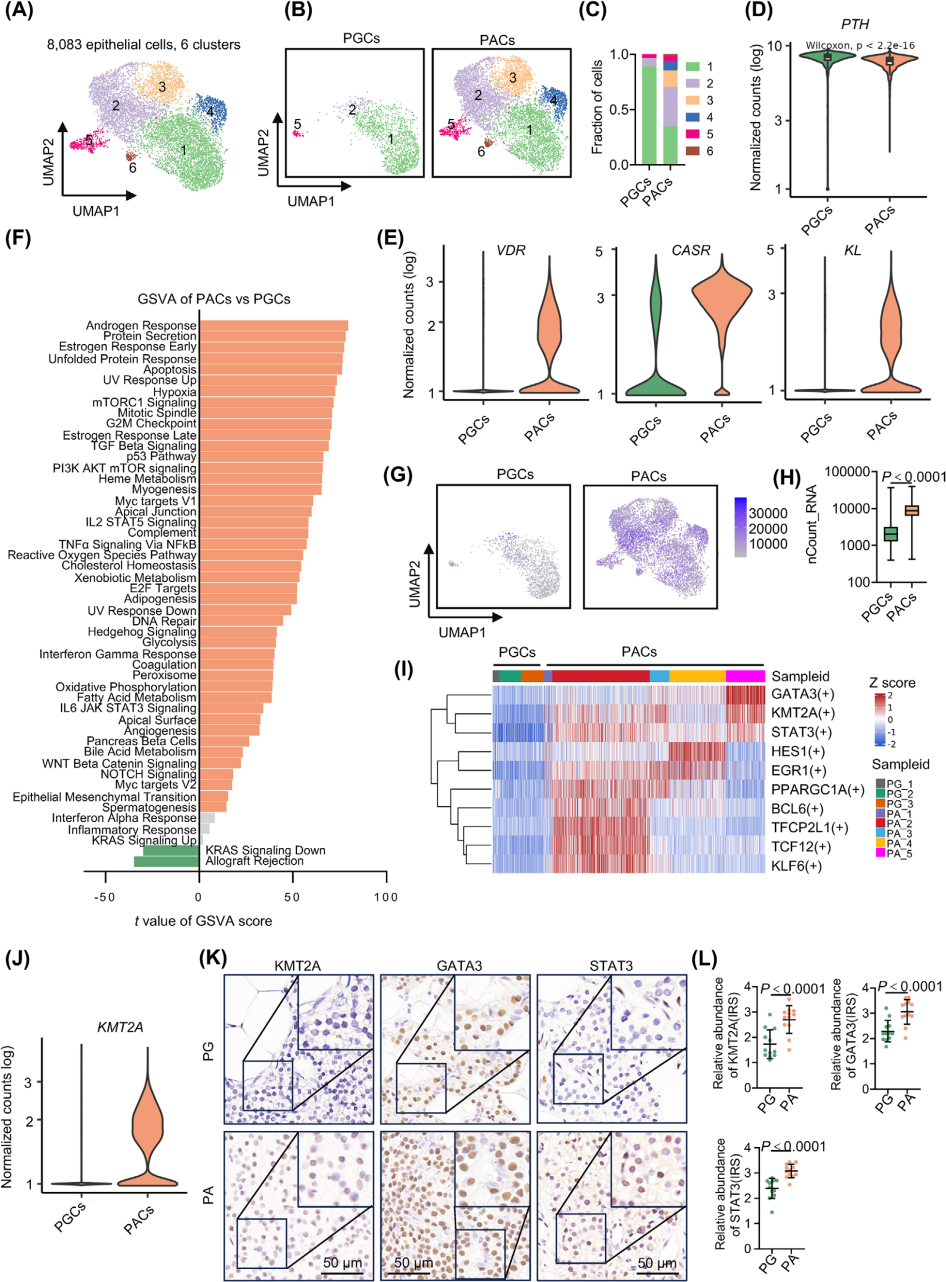
A pervasive increase in transcriptional activity in parathyroid adenoma cells (PACs). (A) UMAP plots of the six epithelial cell clusters with each cell colour‐coded for the associated cluster. (B) UMAPs of the six epithelial cell clusters split by cell types (parathyroid gland cells [PGCs] vs. PACs), colour‐coded by cell cluster. (C) Proportion plots of PGCs and PACs in each cluster as shown in (B). (D and E) Violin plots of the expression distribution of *PTH* (D) as well as *vitamin D receptor* (*VDR*), *calcium‐sensing receptor* (*CASR*) and *Klotho* (*KL*) (E) in PGCs and PACs (*n* = 6589 and 1494 cells for PACs and PGCs, respectively). (F) Differential expression of hallmark pathway gene signatures scored per cell using gene set variation analysis (GSVA) in PACs versus PGCs (*n* = 6589 and 1494 cells for PACs and PGCs, respectively). The data shown are *t* values from a linear model. UV, ultraviolet; v1, version 1; v2, version 2. (G) UMAPs of the six epithelial cell clusters split by cell types (PGCs vs. PACs), colour‐coded according to the number of transcripts (UMIs) detected in each cell. (H) Box plots of UMIs detected in PACs and PGCs (with plot center, box and whiskers corresponding to median, interquartile range [IQR] and 1.5 × IQR, respectively; *n* = 6589 and 1494 cells for PACs and PGCs, respectively). (I) Heatmap of the area under the curve (AUC) scores of the transcription factor binding motifs per PAC relative to per PGC estimated using single‐cell regulatory network inference and clustering (SCENIC). The data shown are the 10 transcription factors exhibiting the most significant differences between a PAC and a PTC. (J) Violin plots of the expression distribution of *KMT2A* in PACs and PGCs (*n* = 6589 and 1494 cells for PACs and PGCs, respectively). (K) Representative microphotographs of immunohistochemistry (IHC) staining of histone‐lysine N‐methyltransferase 2A (KMT2A), GATA binding protein 3 (GATA3) and signal transducer and activator of transcription 3 (STAT3) on formalin‐fixed paraffin‐embedded (FFPE) PA and PG tissue sections. (L) Quantitation of KMT2A, GATA3 and STAT3 expression in PA (*n* = 12) and PG (*n* = 12) tissues as shown in (K). The data shown are the mean immunoreactive score (IRS) ± standard deviation (SD). Two‐tailed Student's *t*‐test.

To understand the molecular properties of PACs compared with PGCs, we further grouped epithelial cells into six clusters (Figure 2A). This analysis revealed that PGCs were mainly located in clusters 1, 2 and 5, while clusters 3, 4 and 6 were composed exclusively of PACs (Figure 2B,C and Table S4), indicating that these PACs possess distinctive transcriptomic characteristics. Indeed, depicting the development trajectory of clusters 1−6 revealed a multifurcating structure with cluster 1 situated opposite the other clusters (Figure S3A), whereas PACs appeared highly heterogeneous, displaying multiple developmental trajectories (Figure S3A,B). Nevertheless, PGCs and PACs exhibit a relatively distinct separation (Figure S3B).

Consistent with previous results,^35^, ^36^ PACs exhibited moderately yet significantly lower expression of *PTH* than PGCs (Figure 2D). In accord with these findings, the overall levels of *CASR*, *vitamin D receptor* (*VDR*) and *Klotho* (*KL*), which are known to negatively regulate PTH production and secretion, were increased in PACs (Figure 2E).^35^, ^36^ The reduction in *PTH* and the increase in *CASR* in PACs were reflected in their protein expression levels, as detected using IHC in independent FFPE PA and PG tissues (Figure S3C‒F). Intriguingly, the expression of *GATA3*, *MAF bZIP transcription factor B* (*MAFB*) and *Glial Cells Missing Transcription Factor 2* (*GCM2*), genes that encode TFs involved in transactivation of *PTH*,^37^, ^38^, ^39^ was increased in PACs (Figure S3G), suggesting that mechanisms beyond a decrease in transcription could be responsible for the reduction in *PTH* mRNA expression. Indeed, genes involved in mRNA catabolism and in regulating RNase activity were increased in PACs compared with PGCs (Figure S3H), proposing that the reduction in *PTH* mRNA levels in PACs may be due to an increase in its degradation.

We then carried out GSVA of hallmark pathway gene signatures,^40^ comparing PACs with PGCs. Strikingly, among the 50 hallmark pathways, 45 were significantly enriched in PACs (Figure 2F), indicating a widespread increase in the expression of genes contributing to these pathways. Consistently, cells in clusters 2‒4, which were exclusively PACs, exhibited increased activation in many hallmark pathways compared with cells in other clusters (Figure S3I). Similar to the developmental trajectory (Figure S3A,B), cells in cluster 1 displayed the least activation of almost all the pathways. Indeed, the average transcript read counts in PACs were approximately 3.5‐fold higher than those in PGCs (Figure 2G,H). In contrast, the average transcript read counts for fibroblasts, myeloid cells, and T and NK cells from PA tissues were lower than those from PG tissues (Figure S3J), suggesting that the increase in transcript read counts in PACs was not due to technical bias. Moreover, the increase was not attributed to reduced RNA degradation (Figure S3H), suggesting that the higher RNA content in PACs results from transcriptional increases. Interestingly, the androgen and estrogen response signatures were among the most enriched pathways (Figure 2F), although both estrogen receptor (ER) genes (*ESR1* and *ESR2*) and the androgen receptor (AR) gene (*AR*) were hardly detectable in PACs (Figure S3K). This suggests that cell‐autonomous ER and AR signalling, which is typically associated with cell proliferation, is likely involved in PA pathogenesis.^41^, ^42^ In contrast, the allograft rejection pathway was less active in PACs (Figure 2F), suggesting that PACs have potentially developed the ability to evade the immune response.^43^

To gain mechanistic insights into the widespread increase in transcription in PACs, we used SCENIC analysis.^44^ This analysis showed that the regulon of KMT2A, a histone methyltransferase positively regulating gene transcription through trimethylating histone H3 at lysine 4 (H3K4), along with the regulons of GATA3 and STAT3, which are involved in regulating cell proliferation, were the most upregulated regulons in PACs compared with PGCs (Figure 2I).^45^, ^46^ Indeed, *KMT2A* and *STAT3* were upregulated in PACs, similar to the upregulation observed for *GATA3* (Figures 2J and S3G). Moreover, IHC analysis demonstrated that the KMT2A, GATA3 and STAT3 proteins were expressed at increased levels in FFPE PA compared to PG tissues (Figure 2K,L). Notably, the gene encoding EZH2 was only scarcely detected in PACs (Figure S3L). Collectively, these results point to the potential involvement of KMT2A‐mediated epigenetic mechanisms in conjunction with GATA3‐ and STAT3‐mediated transcriptional regulation in promoting the pervasive increase in transcriptional activity in PACs.

### 3.3 The KMT2A‒STAT3/GATA3‒cyclin D2 axis may play a role in promoting PAC proliferation

**FIGURE 3:**
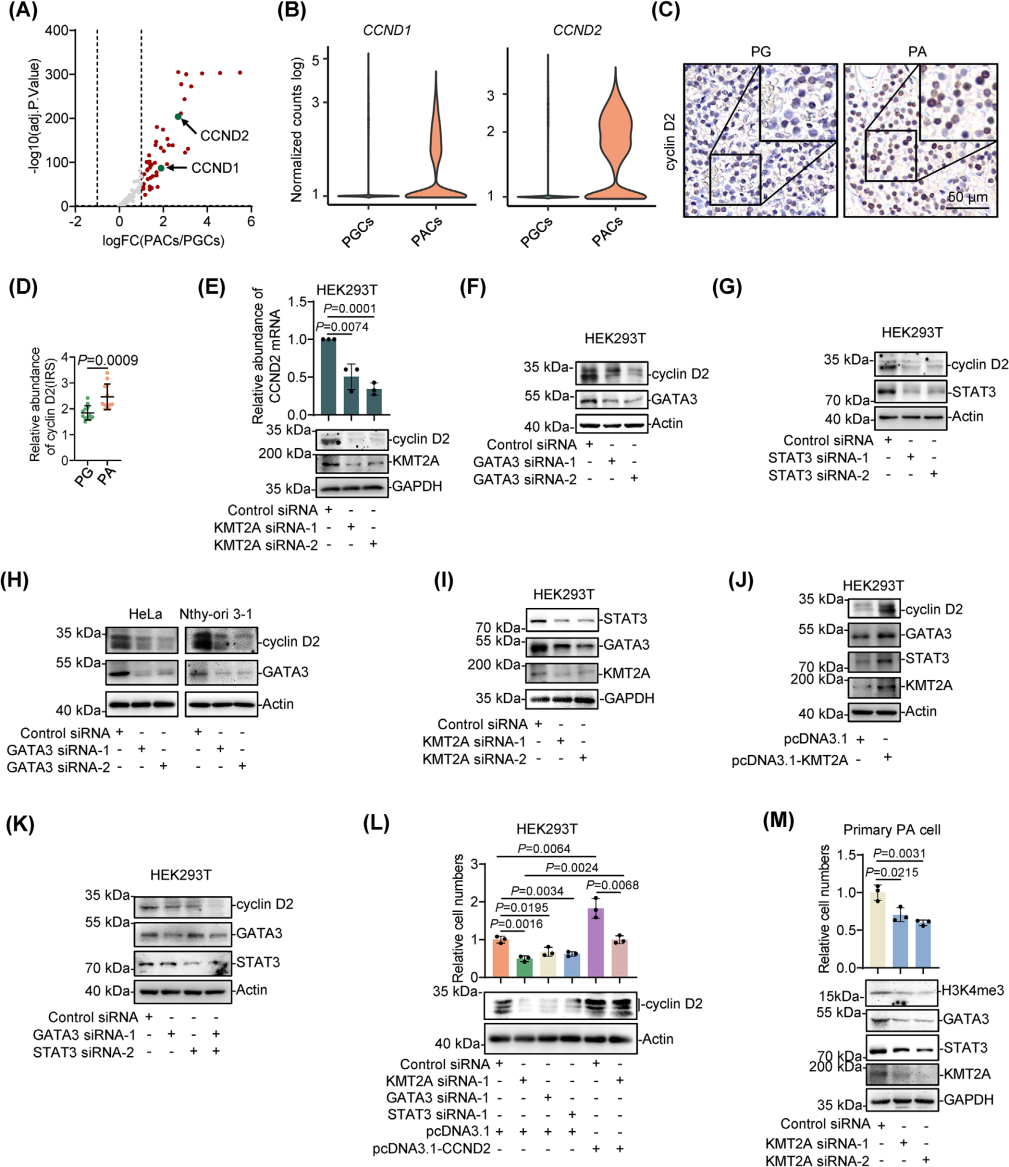
The KMT2A‒STAT3/GATA3‒cyclin D2 axis may play a role in promoting parathyroid adenoma cell (PAC) proliferation. (A) Volcano plot of the differentially expressed genes included in the Kyoto Encyclopedia of Genes and Genomes (KEGG) cell cycle pathway between PACs and parathyroid gland cells (PGCs). (B) Violin plots of the expression distribution of *CCND1* and *CCND2* in PACs and PGCs (*n* = 6589 and 1494 cells for PACs and PGCs, respectively). (C) Representative microphotographs of immunohistochemistry (IHC) staining of cyclin D2 on formalin‐fixed paraffin‐embedded (FFPE) PG and PA tissue sections (*n* = 12). (D) Quantitation of cyclin D2 expression in PA (*n* = 12) and PG (*n* = 12) tissues as shown in (C). Data shown are mean immunoreactive score (IRS) ± standard deviation (SD). Two‐tailed Student's *t*‐test. (E) siRNA knockdown of histone‐lysine N‐methyltransferase 2A (KMT2A) downregulated cyclin D2 expression at the mRNA (upper panel) and protein (lower panel) levels in HEK293T cells. The data are representatives or mean ± SD; *n* = 3 independent experiments, two‐tailed Student's *t*‐test. (F) siRNA knockdown of GATA binding protein 3 (GATA3) downregulated cyclin D2 expression in HEK293T cells. The data are representatives; *n* = 3 independent experiments. (G) siRNA knockdown of signal transducer and activator of transcription 3 (STAT3) downregulated cyclin D2 expression in HEK293T cells. The data are representatives; *n* = 3 independent experiments. (H) siRNA knockdown of GATA3 downregulated cyclin D2 expression in HeLa cells and Nthy‐ori 3‐1 cells. The data are representatives; *n* = 3 independent experiments. (I) siRNA knockdown of KMT2A downregulated GATA3 and STAT3 expression in HEK293T cells. The data are representatives; *n* = 3 independent experiments. (J) KMT2A overexpression upregulated GATA3, STAT3 and cyclin D2 in HEK293T cells. The data are representatives; *n* = 3 independent experiments. (K) Co‐knockdown of GATA3 and STAT3 reduced cyclin D2 expression to a greater extent compared with knockdown of GATA3 or STAT3 alone in HEK293T cells. The data are representatives; *n* = 3 independent experiments. (L) siRNA knockdown of KMT2A, GATA3 or STAT3 suppressed the growth of HEK293T cells, whereas overexpression of cyclin D2 diminished the inhibition of cell proliferation caused by knockdown of KMT2A. The data are representatives or mean ± SD; *n* = 3 independent experiments, two‐tailed Student's *t*‐test. (M) siRNA knockdown of KMT2A downregulated H3K4me3, GATA3 and STAT3 expression and decreased the proliferation of the primary parathyroid adenoma cells. The data are representatives or mean ± SD; *n* = 3 independent experiments.

To test the mechanism responsible for the increased proliferation of PACs more directly, we compared the expression of genes encompassed in the Kyoto Encyclopedia of Genes and Genomes (KEGG) cell cycle pathway between PACs and PGCs.^47^ As anticipated, a series of genes associated with cell cycle progression were upregulated in PACs (Figure 3A). Intriguingly, the gene encoding cyclin D2, *CCND2*, was more prominently upregulated than the gene encoding cyclin D1, *CCND1* (Figure 3A,B), whose increase is known to be a causative factor in a subset of PAs.^48^, ^49^ Further analysis showed that *CCND2* expression was elevated in PACs from all patients, whereas *CCND1* expression in PACs from patient 2 was hardly detectable, similar to normal PGCs (Figure S3M). Substantiating the upregulation of *CCND2*, IHC analysis revealed that cyclin D2 was upregulated in FFPE PA compared with PG samples (Figure 3C,D). Together, these data suggest that the increase in cyclin D2 expression may play a role in promoting proliferation in PACs.

To examine whether cyclin D2 upregulation involves transcriptional increases mediated by KMT2A, GATA3 and STAT3, we carried out siRNA knockdown experiments in HEK293T cells, which have been used by other researchers for experiments in cellular biology, molecular biology and biochemistry related to parathyroid disease studies.^50^, ^51^, ^52^ While knockdown of KMT2A markedly reduced the expression of cyclin D2 at the mRNA and protein levels (Figure 3E), knockdown of GATA3 or STAT3 also decreased the levels of cyclin D2, albeit more moderately (Figure 3F,G). Indeed, bioinformatics analysis revealed that there are four GATA3 consensus binding sites within the promoter region of *CCND2* (Figure S3N). Of note, STAT3 has been previously shown to regulate cyclin D2 expression,^53^ whereas our experiments using HeLa cells and Nthy‐ori 3‐1 human thyroid follicular epithelial cells supported the regulatory role of GATA3 in cyclin D2 expression (Figure 3H).

Interestingly, knockdown of KMT2A led to downregulation of GATA3 and STAT3 (Figure 3I), indicating that GATA3 and STAT3 expression is regulated by KMT2A. In support of this, overexpression of KMT2A resulted in increases in GATA3 and STAT3 as well as cyclin D2 expression (Figure 3J). The simultaneous knockdown of GATA3 and STAT3 reduced cyclin D2 expression to a similar extent as knockdown of KMT2A alone (Figure 3K). Moreover, while knockdown of KMT2A, and to a lesser extent, knockdown of GATA3 and STAT3, reduced cell proliferation, overexpression of cyclin D2 diminished the decrease in proliferation caused by knockdown of KMT2A, GATA3 or STAT3 (Figure 3L). Thus, KMT2A upregulates GATA3 and STAT3, which in turn cooperatively activate CCND2 to promote cell proliferation. Importantly, siRNA knockdown of KMT2A significantly reduced proliferation in a primary PAC culture (Figure 3M). Moreover, consistent with its role as a histone methyltransferase, KMT2A knockdown reduced the expression level of H3K4me3, one of the transcriptional activation marks,^54^ along with GATA3 and STAT3, in the culture (Figure 3M). Together, these results suggested that the KMT2A‒STAT3/GATA3‒cyclin D2 axis may also operate in PACs to promote their proliferation.

### 3.4 Enrichment of proinflammatory fibroblasts in the PA microenvironment

**FIGURE 4:**
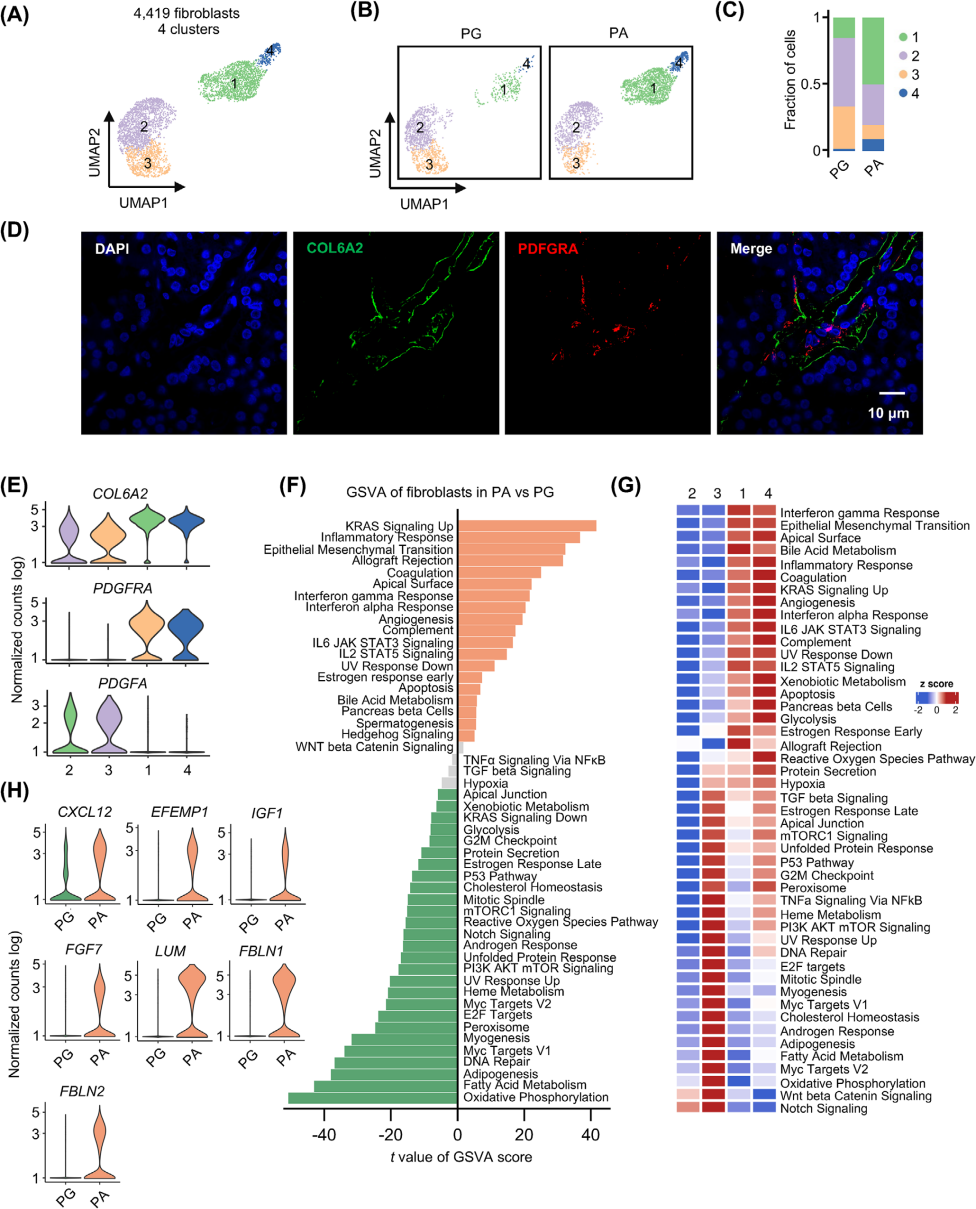
Enrichment of proinflammatory fibroblasts in the parathyroid adenoma (PA) microenvironment. (A) UMAP plots of the four fibroblast clusters with each cell colour coded for the associated cluster. (B) UMAPs of the four fibroblast clusters split by cell types (parathyroid gland [PG] fibroblasts vs. PA fibroblasts), colour coded by cell cluster. (C) Proportion plots of PG and PA fibroblasts in each cluster as shown in (B). (D) Representative microphotographs of immunofluorescence (IF) staining of collagen type VI alpha 2 chain (COL6A2) and platelet‐derived growth factor receptor alpha (PDGFRA) on formalin‐fixed paraffin‐embedded (FFPE) PA (*n* = 3) tissue sections. (E) Violin plots of the expression distribution of *COL6A2*, *PDGFRA* and *PDGFA* in different clusters of fibroblasts (*n* = 1676, 1659, 800 and 284 for clusters 1−4, respectively). (F) Differential expression of hallmark pathway gene signatures scored per cell using gene set variation analysis (GSVA) in PA fibroblasts versus PG fibroblasts (*n* = 2878 and 1541 cells for PA and PG fibroblasts, respectively). The data shown are *t* values from a linear model. UV, ultraviolet; v1, version 1; v2, version 2. (G) Heatmap of the differential expression of hallmark pathway signature genes scored per cell using GSVA among different fibroblast clusters. The data shown are *z* scores from a liner model. (H) Violin plots of the expression distribution of *C‐X‐C Motif Chemokine Ligand 12* (*CXCL12*), *EGF‐containing fibulin‐like extracellular matrix protein 1* (*EFEMP1*), *insulin‐like growth factor 1* (*IGF1*), *fibroblast growth factor 7* (*FGF7*), *lumican* (*LUM*), *fibulin 1* (*FBLN1*) and *fibulin 2* (*FBLN2*) in PA and PG fibroblasts (*n* = 2878 and 1541 cells for PA and PG fibroblasts, respectively).

To investigate the potential importance of the microenvironment in PA pathogenesis, we analysed the transcriptomic profiles of stromal cells of PA in comparison to those from PG tissues. Notably, while the frequency of fibroblasts in PA and PG tissues remained largely comparable (Figure 1C), further analysis grouped the 4419 fibroblasts into four distinct clusters. This grouping revealed that the proportions of clusters 1 and 4 increased, whereas those of clusters 2 and 3 decreased, in PA relative to PG samples (Figure 4A‒C). These findings suggested that PA fibroblasts are characteristically skewed away from clusters 2 and 3 and toward clusters 1 and 4. We employed immunofluorescence (IF) staining of independent FFPE PA tissues, which confirmed the presence of platelet‐derived growth factor receptor alpha (PDGFRA)^+^ and collagen type VI alpha 2 chain (COL6A2)^+^ fibroblasts (Figure 4D). Interestingly, PDGFRA^+^ and COL6A2^+^ fibroblasts appeared to be mainly located in clusters 1 and 4 (Figure 4E). However, they did not appear to be selectively positioned in any clusters of the recently described pan‐cancer fibroblasts,^55^ suggesting that these characteristics of fibroblasts in clusters 1 and 4 are specific to PA tissues.

Intriguingly, a comparison of hallmark pathway gene signatures between fibroblasts from PA and those from PG tissues showed that the inflammatory_response pathway, along with other pathways involved in chronic inflammation, such as the IL6_JAK_STAT3, IFNα_response and angiogenesis pathways, was enriched in PA fibroblasts (Figure 4F). In accordance, fibroblasts in clusters 1 and 4 that primarily consisted of those from PA tissues displayed increased activation of a range of pathways associated with chronic inflammation (Figure 4G). This enrichment suggests that PA fibroblasts are akin to the previously described inflammatory cancer‐associated fibroblasts.^56^, ^57^, ^58^ Indeed, PA fibroblasts exhibited higher expression levels of a number of inflammation‐related genes, including *C‐X‐C Motif Chemokine Ligand 12* (*CXCL12*) and *EGF‐containing fibulin‐like extracellular matrix protein 1* (*EFEMP1*) (Figure 4H). Moreover, the KRAS_signalling pathway, known for promoting the production of growth factors, chemokines and extracellular matrix proteins was also found to be significantly enriched in PA fibroblasts (Figure 4F).^59^ Similarly, PA fibroblasts showed markedly higher levels of *insulin‐like growth factor 1* (*IGF1*) and *fibroblast growth factor 7* (*FGF7*) compared to PG fibroblasts (Figure 4H). Of note, certain extracellular matrix protein‐encoding genes, including *lumican* (*LUM*), *fibulin 1* (*FBLN1*) and *fibulin* 2 (*FBLN2*), were expressed in PA but were hardly detectable in PG fibroblasts (Figure 4H), further highlighting the functional dissimilarities between the two fibroblast types. Interestingly, alongside the high expression of *PDGFRA* that encodes a tyrosine kinase receptor in clusters 1 and 4, the gene for the PDGFRA ligand, *platelet‐derived growth factor subunit A* (*PDGFA*), was predominantly detected in clusters 2 and 3 (Figure 4E),^60^ suggesting that fibroblasts in clusters 2 and 3 might influence the proliferation and growth of those in clusters 1 and 4 by secreting PDGFA. Collectively, these data suggest that the PA microenvironment is characterised by an abundance of proinflammatory fibroblasts, which may play a role in the pathogenesis of the disease.

### 3.5 ECs in PAs display a proinflammatory signature

**FIGURE 5:**
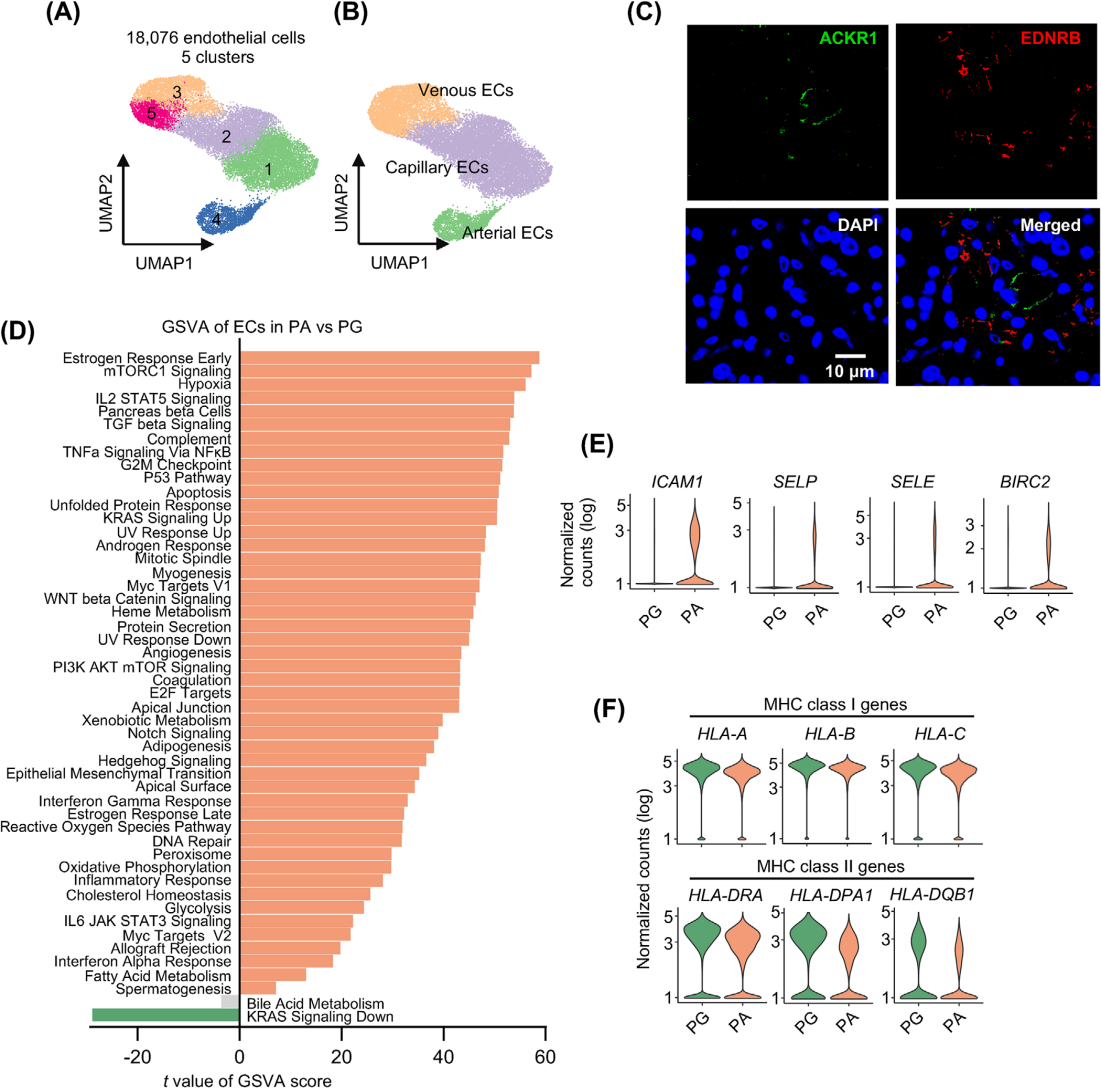
Endothelial cells (ECs) in parathyroid adenomas (PAs) display proinflammatory signatures. (A) UMAP plots of the five EC clusters with each cell colour coded for the associated cluster. (B) UMAPs of the three EC types, colour coded for venous EC, capillary ECs and arterial ECs. (C) Representative microphotographs of immunofluorescence (IF) staining of atypical chemokine receptor 1 (ACKR1) and endothelin receptor type B (EDNRB) on formalin‐fixed paraffin‐embedded (FFPE) PA (*n* = 3) tissue sections. (D) Differential expression of hallmark pathway gene signatures scored per cell using gene set variation analysis (GSVA) in PA versus parathyroid gland (PG) ECs (*n* = 7352 and 10 724 cells for PG and PA ECs, respectively). The data shown are *t* values from a linear model. UV, ultraviolet; v1, version 1; v2, version 2. (E) Violin plots of the expression distribution of *intercellular adhesion molecule 1* (*ICAM1*), *selectin P* (*SELP*), *selectin E* (*SELE*) and *baculoviral IAP repeat‐containing protein 2* (*BIRC2*) in PA and PG ECs (*n* = 7352 and 10 724 cells for PG and PA ECs, respectively). (F) Violin plots of the expression distribution of the major histocompatibility complex (MHC) class I genes (*HLA‐A*, *HLA‐B* and *HLA‐C*) and MHC class II genes (*HLA‐DRA*, *HLA‐DPA1* and *HLA‐DQB1*) in PA and PG ECs (*n* = 7352 and 10 724 cells for PG and PA ECs, respectively).

With 18 076 cells detected in our analysis, ECs represented the most abundant cell type (Figures 1A‒C and 5A), consistent with the rich vasculature of PG and PA tissue.^61^ Subsequent re‐clustering revealed five subclusters of blood vessel ECs, designated as capillary ECs (clusters 1 and 2), arterial ECs (cluster 4) and venous ECs (clusters 3 and 5) (Figures 5A,B and S4A,B). The presence of atypical chemokine receptor 1 (ACKR1)^+^ venous and endothelin receptor type B (EDNRB)^+^ capillary ECs was confirmed using IF staining of independent FFPE PA tissue sections (Figure 5C).

GSVA revealed that all but two pathways were significantly enriched in PA ECs compared with PG ECs (Figure 5D), suggesting a stronger transcriptional activation status of PA ECs. Consistent with this observation, the total transcript read counts per PA EC were ∼2‐fold higher than per PG EC (Figure S4C). Of note, ECs in cluster 5, which originated primarily from PA tissues, displayed increased activation of many hallmark pathways compared with those in the other clusters (Figure S4D). Interestingly, in contrast to the often downregulation of the inflammatory_response pathway in ECs in the microenvironment of malignant tumours,^62^, ^63^ several signatures involved in inflammation and the immune_response pathway were among the most upregulated pathways in PA ECs, including the IL2_STAT5 and TNFα_signalling_via_NFκB pathways (Figure 5D). Consistently, *intercellular adhesion molecule 1* (*ICAM1*), and to a lesser extent, *selectin P* (*SELP*) and *selectin E* (*SELE*), which mediate immune cell extravasation, were expressed at higher levels in PA than PG ECs (Figure 5E). Similarly, the expression of *baculoviral IAP repeat‐containing protein 2* (*BIRC2*) that is involved in the innate immune response was increased in PA ECs (Figure 5E). Moreover, unlike the frequent downregulation of *major histocompatibility complex* (*MHC*) *class I* and *II* genes essential for antigen presentation and activation of the adaptive immune system in ECs evident in malignant tumour tissues,^62^, ^63^ MHC class I genes such as *human leukocyte antigen A* (*HLA‐A*), *HLA‐B* and *HLA‐C* and MHC class II genes such as *HLA‐DRA*, *HLA‐DPA1* and *HLA‐DQB1* were expressed at comparable levels in PA and PG ECs (Figure 5F). The high expression of MHC class I and II genes was not due to inadvertent doublets, as we detected very few marker genes associated with various cell types, including immune cells, within the EC population (Figure 1B). Thus, unlike ECs in the malignant environment,^62^ the ECs in PA tissues may maintain their intact functionality and even possess augmented abilities to facilitate inflammation and immune responses.

### 3.6 Enrichment of macrophages with rheostatic phenotypes in PAs

**FIGURE 6:**
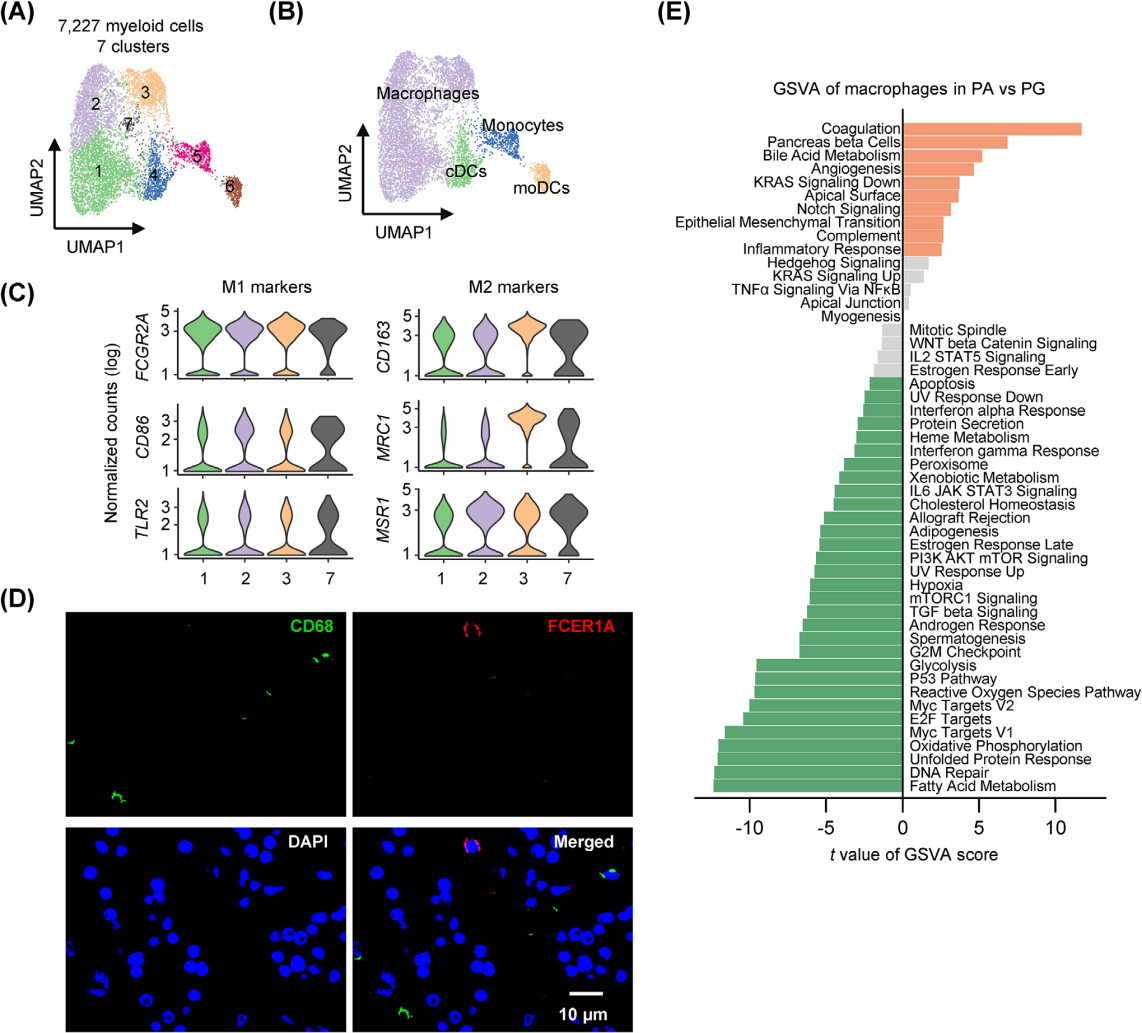
Enrichment of macrophages with rheostatic phenotypes in parathyroid adenomas (PAs). (A) UMAP plots of the seven myeloid cell clusters with each cell colour coded for the associated cluster. (B) UMAPs of the four types of myeloid cells, colour‐coded for macrophages, monocytes, conventional dendritic cells (cDCs) and monocyte‐derived dendritic cells (moDCs). (C) Violin plots of the expression distribution of the M1 marker genes (*FCGR2A*, *CD86* and *TLR2*) and M2 marker genes (*CD163*, *MRC1* and *MSR1*) in PA and parathyroid gland (PG) macrophages (*n* = 2693, 1788, 1031 and 361 cells for clusters 1, 2, 3 and 7, respectively). (D) Representative microphotographs of immunofluorescence (IF) staining of CD68 and Fc epsilon receptor IA (FCER1A) on formalin‐fixed paraffin‐embedded (FFPE) PA (*n* = 3) tissue sections. (E) Differential expression of hallmark pathway gene signatures scored per cell using gene set variation analysis (GSVA) in PA versus PG macrophages (*n* = 774 and 4859 cells for PA and PG macrophages, respectively). The data shown are *t* values from a linear model. UV, ultraviolet; v1, version 1; v2, version 2.

The frequency of myeloid cells was higher in PA than in PG tissues (Figure 1C), implicating their potential involvement in PA pathogenesis. To gain deeper insights, we grouped the total of 7227 myeloid cells into seven clusters (Figure 6A). One cluster corresponded to monocytes (cluster 5), and two clusters to dendritic cells (DCs): conventional DCs (cDCs) (cluster 4) and monocyte‐derived DCs (moDCs) (cluster 6), whereas other clusters (clusters 1, 2, 3 and 7) were designated as macrophages (Figures 6B and S5A). Interestingly, the clusters of macrophages (clusters 1, 2, 3 and 7) displayed a poor separation with one another (Figure 6A,B), implying that they may signify different cell statuses on a graded scale, rather than different cellular entities.^62^, ^64^ This is consistent with the macrophage activity spectrum model.^62^, ^64^ Accordingly, macrophage states did not follow the M1 and M2 polarisation model, as M1 and M2 macrophage marker genes—for example, the M1 markers *Fc gamma receptor 2A* (*FCGR2A*), *CD86* and *Toll‐like receptor* (*TLR2*) and the M2 markers *CD163*, *mannose receptor c‐type 1* (*MRC1*) and *macrophage scavenger receptor 1* (*MSR1*)—were co‐expressed by macrophages across all clusters (Figure 6C).^62^, ^63^, ^65^ For verification, we substantiated the infiltration of CD68^+^ macrophages and Fc epsilon receptor IA (FCER1A)^+^ cDCs in independent PA tissues using IF staining (Figure 6D).

GSVA uncovered that numerous pathways, comprising fatty acid metabolism, oxidative phosphorylation and glycolysis, which are essential for energy production and biosynthesis, were downregulated in PA macrophages compared with those derived from PGs (Figure 6E). This suggests a reduction in the overall transcriptional activity. Indeed, the total transcript read counts in each PA macrophage were significantly lower than those in PG macrophages (Figure S5B). Moreover, the total transcript read counts in each monocyte, cDC and moDC from PAs were similarly reduced compared with those from PGs (Figure S5B), indicating that the transcriptomes of myeloid cells are generally repressed in the PA microenvironment. Nevertheless, the inflammatory_response pathway was enriched, albeit moderately, in PA macrophages (Figure 6E), indicating a proinflammatory phenotype.

### 3.7 Transcriptional repression of T cells and NK cells in the PA microenvironment

**FIGURE 7:**
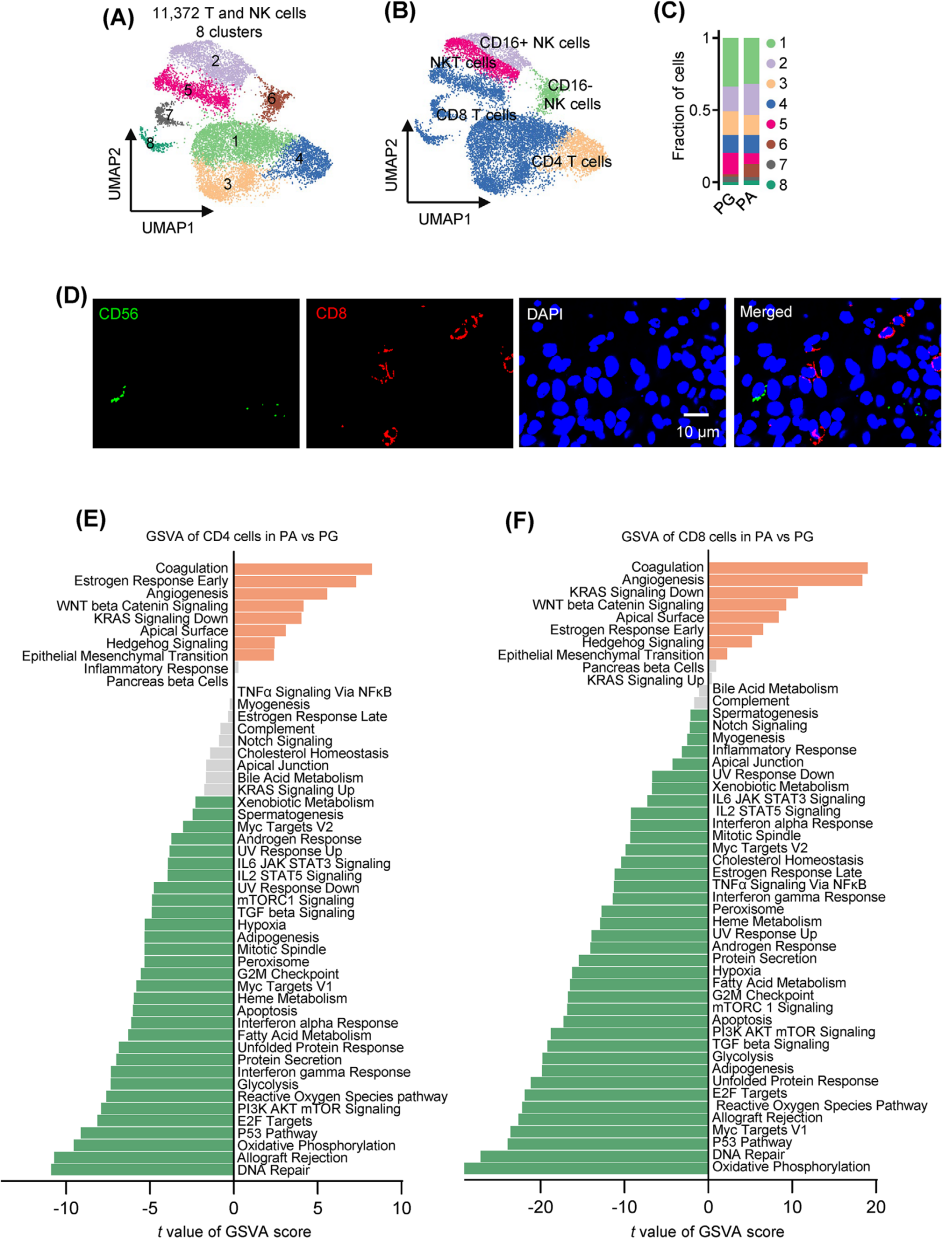
Transcriptional repression in parathyroid adenoma (PA)‐derived T cells and natural killer (NK) cells. (A) UMAP plots of the eight T and NK‐cell clusters with each cell colour coded for the associated cluster. (B) UMAPs of the five types of T and NK cells, colour coded for CD8+ T cells, CD4+ T cells, CD16^+^ NK cells, CD16^‒^ NK cells and NKT cells. (C) Proportion plots of the eight clusters of T and NK cells derived from parathyroid gland (PG) and PA tissues as shown in (A) and (B), respectively. (D) Representative microphotographs of immunofluorescence (IF) staining of CD56 and CD8 on formalin‐fixed paraffin‐embedded (FFPE) PA (*n* = 3) tissue sections. (E and F) Differential expression of hallmark pathway gene signatures scored per cell using gene set variation analysis (GSVA) in PA versus PG CD4 (E; *n* = 737 and 679 cells for PA and PG CD4 T cells, respectively) and CD8 (F; *n* = 2918 and 2416 cells for PA and PG CD8+ T cells, respectively). The data shown are *t* values from a linear model. UV, ultraviolet; v1, version 1; v2, version 2.

Altogether, we identified 11 372 T and NK cells, which were further classified into eight clusters (Figures 1A and 7A). Clusters 1, 3, 5, 7 and 8 were designated as CD8^+^ T cells (*CD8A* ^+^), cluster 4 as CD4^+^ T cells (CD4^+^) and cluster 6 as NK cells (neural cell adhesion molecule 1 [*NCAM1*]^+^CD3D^−^), whereas cluster 2 contained both NK cells and NKT cells (*NCAM1* ^+^CD3D^+^) (Figures 7A,B and S6A). Cells from all PA and PG samples occupied all clusters, although the relative proportion from each sample in each cluster varied (Figures 7C and S6B). We verified the presence of CD8^+^ T cells and CD56^+^ (encoded by *NCAM1*) NK cells using IF staining of independent FFPE PA tissues (Figure 7D).

Strikingly, GSVA revealed widespread reductions in many pathways critically involved in the immune response in PA‐derived T cells, NK cells and NKT cells compared with the counterparts from PGs, including the allograft_rejection pathways (Figures 7E,F and S6C,D). This implicates compromised immune functions of these cells. Moreover, there were decreases in numerous other pathways, such as oxidative_phosphorylation and glycolysis pathways (Figures 7E,F and S6C,D), suggesting broad suppression of transcriptional activity. In line with this, the total transcript read counts in each CD8^+^ T cell, CD4^+^ T cell, NK cell and NKT cell derived from PAs were lower than those from PGs (Figure S6E). Nevertheless, we noticed that the angiogenesis pathway was commonly enriched in PA T and NK cells (Figures 7E,F and S6C,D), suggesting that these cells may contribute to regulating vasculature in PAs.

### 3.8 Cell‐to‐cell interactions within the PA microenvironment potentially promote tumourigenesis

**FIGURE 8:**
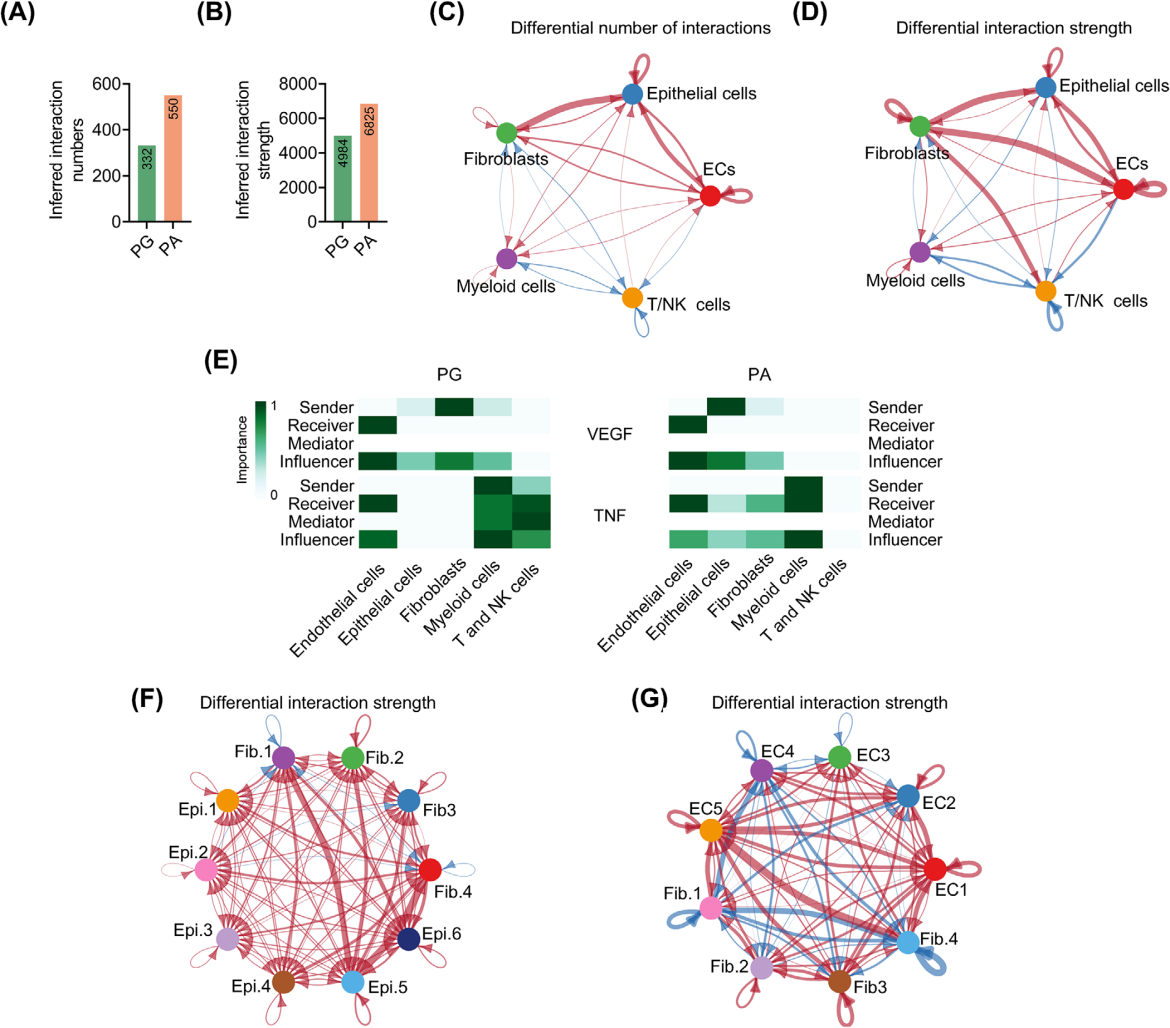
Cell‐to‐cell interactions within the parathyroid adenoma (PA) microenvironment. (A and B) Comparison of the inferred interaction numbers (A) and strength (B) among different cell types in PA and parathyroid gland (PG) microenvironments. (C and D) Circos plots showing intercellular interaction numbers (left) and strength (right) predicted on the basis of known ligand‒receptor pairs among different cell types in PG and PA microenvironments. The strings are directional and represent interaction numbers (C) and strength (D) determined on the basis of the expression of a ligand by one cell type and the expression of the corresponding receptor by another cell type. The thickness of each string corresponds to the numbers (C) or the strength weights (D) of the interaction pairs. The dot colour was coded for the associated cell types. Red lines indicate increased numbers or strength in the PA relative to PG microenvironment, while blue lines signify reduced numbers or strength in the PA versus PG microenvironment. (E) Heatmaps showing the relative importance based on the computed four network centrality measures (signalling sources, targets, mediators and influencers) of the vascular endothelial growth factor A (VEGFA) and tumour necrosis factor (TNF) signalling networks in PG (left) and PA (right) tissues. (F and G) Circos plots showing intercellular interaction strength predicted on the basis of known ligand‒receptor pairs among fibroblast and epithelial cells (F) or fibroblast and endothelial cells (ECs) (G) in PG and PA microenvironments. The strings are directional and represent interaction strength determined on the basis of the expression of a ligand by one subcluster and the expression of the corresponding receptor by another subcluster. The thickness of each string corresponds to the strength weights of the interaction pairs. The dot colour was coded for the associated cell types. Red lines indicate increased strength in the PA relative to PG microenvironment, while blue lines signify reduced numbers or strength in the PA versus PG microenvironment. Epi.: epithelial cells; Fib.: fibroblast.

To compare the global intercellular communications between PA and PG tissues, we employed CellChat, which analyses major signalling inputs and outputs of cells and predicts how the cells and signals coordinate for biological functions.^31^ This analysis inferred that both the numbers and strength of intercellular interactions were increased in PAs compared with PGs (Figure 8A,B). In particular, PA fibroblasts exhibited enhanced outgoing signals directed towards PACs and ECs (Figure 8C,D), implying that fibroblasts may have a part in modulating the biological properties of PACs and in activating ECs (Figures 4F and 5D). Although the number of outgoing interactions from PA fibroblasts to T/NK cells was reduced in PA tissues, the strength of the remaining signals was markedly increased, suggesting that these signals may contribute to the suppression of transcriptional activity in the T and NK cells (Figures 7, 8 and S6C,D). Moreover, the reduced interactions between myeloid cells and T/NK cells, along with the decreased auto‐interactions among T/NK cells were conceivably involved (Figure 8C,D). There were increased reciprocal interactions between ECs and PACs compared with those between ECs and PGCs (Figure 8C,D), signalling the importance of the vasculature in PA pathogenesis.

A more detailed analysis of cell‐to‐cell communication patterns revealed that PACs, instead of fibroblasts, were the primary senders of vascular endothelial growth factor (VEGF) signalling in the PA microenvironment, mainly targeting ECs (Figure 8E). This supports a strong stimulatory effect of PACs on angiogenesis. In contrast to PGCs, PACs received tumour necrosis factor (TNF) signalling (Figure 8E), aligning with the notion that chronic inflammation affects the pathological behaviors of PACs. Moreover, unlike PG fibroblasts, PA fibroblasts received TNF signalling as well (Figure 8E), suggesting that they, too, are subject to regulation by chronic inflammation.

As fibroblasts sent strong outgoing signals to epithelial cells and ECs (Figure 8D), we conducted further analyses on the interactions between subclusters of fibroblasts and epithelial cells, as well as ECs. The results revealed that fibroblasts from clusters 1 and 4 were the major source of the outgoing signals to epithelial cells, with epithelial cells in cluster 5 being the primary targets (Figure 8F). Notably, fibroblasts in clusters 1 and 4, which were mainly derived from PA tissues, displayed the characteristics of inflammatory cancer‐associated fibroblasts (Figure 4G), whereas epithelial cells in cluster 5 exhibited activation of a series of inflammatory pathways (Figure S3I), suggesting that PA fibroblasts may stimulate chronic inflammatory responses in PACs. On the other hand, fibroblasts in cluster 4 were found to be the major contributors of signals to ECs, with ECs in cluster 5 being the major receivers of these signals (Figure 8G). ECs in cluster 5 are characterised by the activation of most pathways (Figure S4D), implying that PA fibroblasts in cluster 4 may play an important role in activating ECs.

## 4 DISCUSSION

Herein, we present a unique single‐cell transcriptomic atlas detailing the cellular composition of PA and PG tissues. The identification of transcriptomic profiles of individual cell types has permitted characterisation of the differences that exist between PAs and PGs. Foremost, we revealed a pervasive transcriptional upregulation in PACs compared with PGCs involving a plethora of genes. Moreover, we highlighted the potential role of the KMT2A‒STAT3/GATA3‒cyclin D2 axis in promoting PAC proliferation. Additionally, we characterised the extensive infiltration of immune cells into the PA microenvironment and revealed the proinflammatory properties of fibroblasts, ECs and macrophages in PA tissues, thus proposing chronic inflammation as a potential mechanism involved in PA pathogenesis.

As the major cause of pHPT, the aetiology of PA remains incompletely understood.^1^, ^2^ Although epigenetic mechanisms, including DNA methylation and histone modifications, are increasingly appreciated to play important roles in the pathogenesis of many other types of tumours, they have also been observed in some PAs.^6^, ^15^, ^16^ In particular, the upregulation of the histone methyltransferase EZH2 promotes PAC proliferation through activation of the Wnt/β‐catenin pathway.^18^, ^19^ However, the involvement of the *EZH2* gene did not feature in our study. A possible explanation for this discrepancy could be that not all PACs express *EZH2*,^19^, ^20^ whereas, as a caveat, our scRNA‐seq analysis was conducted on a relatively small number of PA cases. Indeed, a previous study showed that there was no statistically significant difference in *EZH2* mRNA levels between pHPT tumours and PG tissues when measured using qPCR.^19^ Regardless of the role of *EZH2* in PA pathogenesis, we uncovered evidence for the involvement of an alternative epigenetic regulator, namely, KMT2A. The regulon of KMT2A was among the most activated signatures in PACs compared with PGCs, suggesting that epigenetic regulation of gene expression by KMT2A may play an important role in PA pathogenesis.

Unlike EZH2, which mainly acts as a transcriptional repressor through catalyzing trimethylation of H3 at K27, leading to heterochromatin formation and thus restricting access of TFs to gene promoters,^19^, ^20^, ^21^ KMT2A functions as a transcriptional coactivator.^66^ It catalyses trimethylation of H3 at lysine 4, resulting in euchromatin to facilitate transactivation of genes.^66^, ^67^ Therefore, the increase in KMT2A expression could be involved in the pervasive increase in gene transcription in PACs. Inappropriate activation of KMT2A, often due to chromosomal rearrangements, contributes to the development and progression of acute lymphoid and myeloid leukaemias.^68^, ^69^ However, this does not necessarily correlate with an increase in KMT2A expression. Notably, genomic amplification has been reported as a cause of EZH2 upregulation in PACs,^19^, ^20^, ^21^ and a somatic missense mutation of the *EZH2* gene has been described to lead to increased EZH2 expression.^16^ Nonetheless, further mechanistic investigations are required to determine the underlying mechanisms of KMT2A upregulation in PACs.

What is the downstream effector potentially involved in KMT2A‐mediated promotion of PA pathogenesis? Our results suggested an important role for the proto‐oncogene *CCND2*, which was upregulated in PACs compared to PGCs to a much greater extent than *CCND1*.^70^, ^71^ The latter is known to be overexpressed in approximately 20%−40% of PAs and is considered a driver of PA development and progression.^6^, ^8^, ^9^, ^48^ The proteins encoded by CCND1 and CCND2, cyclin D1 and cyclin D2 respectively, function similarly to bind and cause activation of cyclin‐dependent kinase 4 (CDK4) and/or 6 (CDK6), driving the transition of cells from G1 to S phase.^70^, ^71^ Therefore, it is likely that the increase in *CCND2* similarly plays a driving role in PA pathogenesis. The upregulation of *CCND1* in PACs is commonly due to chromosomal translocation or gene amplification,^48^, ^72^ but the cause of increased CCND2 expression requires further study. Regardless, our results suggested the possible involvement of GATA3 and STAT3 in transcriptional activation of *CCND2*. GATA3 plays an important role in embryonic development of PG,^27^ whereas STAT3 promotes cell proliferation through direct transcriptional regulation of gene expression or indirect effects via c‐Myc.^73^, ^74^ Indeed, our mechanistic investigations, albeit performed in cells other than PACs and PGCs, demonstrated that GATA3 and STAT3 cooperatively regulate *CCND2* to promote proliferation. Moreover, we found that the expression of both GATA3 and STAT3 is under the control of KMT2A, reinforcing the potential of the KMT2A‒GATA3/STAT3‒cyclin D2 axis in promoting proliferation of cells. Noticeably, we could not validate the upregulation of *KMT2A*, *GATA3* and *STAT3* in PACs using publicly available bulk transcriptome datasets.^35^, ^75^ Nevertheless, this does not negate the findings from our scRNA‐seq analysis, which specifically compared the expression of genes between PACs and PGCs—a comparison that cannot be achieved with bulk cell sequencing.

Chronic inflammation plays an important role in tumourigenesis through multiple mechanisms, such as production of cytokines and growth factors to promote cell survival and proliferation, induction of oxidative stress leading to DNA, protein and lipid damage, promotion of angiogenesis, and inhibition of the anti‐tumour immune response.^76^, ^77^ However, whether it is involved in PA pathogenesis remains unclear, although the infiltration of immune cells into the PA microenvironment has been well documented.^28^, ^78^ Our analysis of stromal cells revealed that the PA microenvironment is markedly more proinflammatory than that of PG tissues. First, PA fibroblasts displayed primarily a phenotype similar to inflammatory cancer‐associated fibroblasts^57^, ^58^; second, ECs in the PA microenvironment were strongly activated, expressing a range of genes necessary for immune cell extravasation; third, the frequency of myeloid cells was markedly higher in PA relative to PT tissues, and the inflammatory_response pathway was upregulated in PA macrophages; fourth, the angiogenesis pathway was enriched in T and NK cells from PAs compared with those from PGs, indicative of the inflammatory response,^79^, ^80^ although numerous hallmark gene signature pathways were downregulated, and last, PACs and PA fibroblasts, in contrast to PGCs and PG fibroblasts, received TNF signalling. The potential importance of proinflammatory fibroblasts in PA pathogenesis was also signified by their enhanced outgoing signals directed to PACs and ECs. Intriguingly, there was widespread repression of gene transcription in T/NK cells in the PA microenvironment, indicating a compromised immune response. Although the underlying mechanisms for this repression remain to be clarified, the strength of a proportion of signalling from PA fibroblasts to T/NK cells was enhanced, whereas the crosstalk between myeloid cells and T/NK cells was reduced, implicating possible roles of proinflammatory fibroblasts and myeloid cells in suppressing T/NK cells in the PA microenvironment. Regardless, our current data suggest that chronic inflammation may contribute to PA pathogenesis, although further investigations are needed to substantiate this.

Of note, while our H&E staining supported the well‐documented observation that the proportion of adipocytes is commonly reduced in PA compared with PG tissues,^81^ we did not identify any clusters of cells that expressed adipocyte marker genes such as *CIDEA*, *PLIN1*, *PPARGC1A* and *ADIPOQ* in our single cell‐sequencing data. This loss of adipocytes could conceivably be caused by the tissue dissociation and cell preparation process. Irrespectively, our data propose an updated model for the pathogenesis of PAs: KMT2A‐mediated epigenetic mechanisms drive a pervasive transcriptional increase in gene expression, leading to the upregulation of STAT3 and GATA3, which in turn transactivate *CCND2* to promote PAC proliferation. Concurrently, a chronic inflammatory microenvironment promotes PA pathogenesis through mechanisms such as angiogenesis and immune suppression (Figure S7).

Nevertheless, many questions remain to be addressed. For example, what is the cause‐and‐effect relationship between KMT2A upregulation in PACs and the chronic inflammation in the PA microenvironment? What are the mechanisms of the dysregulation of gene expression in PA stromal cells? Moreover, given the highly heterogeneous nature of PAs,^28^, ^78^ the relatively small number of PA cases included in this study leaves open the question of how representative the identified mechanisms are. Investigation in larger clinical cohorts is clearly desirable to further validate our findings. Should the proposed model be substantiated experimentally and clinically, it would facilitate the development of novel therapeutic strategies targeting the mechanisms involved in PA pathogenesis. It is worth noting that MM‐401, a small molecule inhibiting the methyltransferase activity of KMT2A, is already in preclinical development.^82^, ^83^

## AUTHOR CONTRIBUTIONS

Feng‐Min Shao, Huixia Cao and Xu Dong Zhang conceived and designed the work. Qin Xu, Liu Teng and Kaihong Ye performed the sample processing. Ting La, Li Wang, Jinming Li and Kaihong Ye established the analytical strategy. Qin Xu and Liu Teng analysed the data. Shasha Wang, Lei Yan, Zhenhua Zhang, Zehua Shao, Xiao Hong Zhao, Yu Chen Feng, Lei Jin and Mark Baker assisted with data analysis and performed experiments. Qin Xu and Huixia Cao identified and gained written informed consent from participating patients. Xu Dong Zhang, Ting La, Huixia Cao and Feng‐Min Shao supervised the study. Xu Dong Zhang, Ting La, Feng‐Min Shao and Rick F. Thorne wrote the manuscript. All authors commented on the manuscript.

## CONFLICT OF INTEREST STATEMENT

The authors declare they have no conflicts of interest.

## ETHICS STATEMENT

Studies using human tissues were approved by the Human Research Ethics Committee of Henan Provincial People's Hospital (2020‐026). Written informed consent was obtained from all patients prior to participation.

## Supporting information

Supporting Information

## Data Availability

The scRNA‐seq data generated in this study are publicly available in Gene Expression Omnibus at GSE190773. All other underlying data for the manuscript are available upon request from the corresponding author X.D.Z.
