# Supplementary material for: KMT2A and chronic inflammation as potential drivers of sporadic parathyroid adenoma
Source: Clin Transl Med. 2024 Jun 18;14(6):e1734. doi: 10.1002/ctm2.1734 (PMC11185127; doi:10.1002/ctm2.1734)
Supplement: Supplementary file 1 — Supporting Information [file CTM2-14-e1734-s001.docx]

**Table S1. List of reagents**

| **Reagent** | **Catalogue No.** | **Company** |
| --- | --- | --- |
| DAPI | C1005 | Beyotime (Shanghai, China) |
| anti-fluorescence quenching reagent | P0176-3 | Beyotime (Shanghai, China) |
| Tissue storage solution | 130-100-008 | Miltenyi Biotec (North Rhine-Westphalia, Germany) |
| Trypsin/EDTA Solution (TE) | R001100 | Thermo Fisher (Waltham, Massachusetts) |
| Collagenase II for cell biology | 2275MG100 | Biofroxx (Einhausen, Germany) |
| UltraPure. Bovine Serum Albumin (BSA, 50 mg/ml) | AM2612 | Thermo Fisher (Waltham, Massachusetts) |
| Red Blood Cell Lysis Solution | 130-094-183 | Miltenyi Biotec (North Rhine-Westphalia, Germany) |
| ProLong™ Glass Antifade Mountant with NucBlue™ Stain | P36981 | Thermo Fisher (Waltham, Massachusetts) |
| RNA/DNA extraction kit | R0017M | Beyotime (Shanghai, China) |
| ReverTra Ace qPCR RT Kit | FSQ-101 | TOYOBO (OSAKA JAPAN) |
| 2×RealStar Fast SYBR qPCR Mix | A301-05 | GenStar (Beijing, China) |
| PAGE Gel Kit | G2043 | Servicebio (Wuhan, China) |
| PMSF | P0100 | Solarbio (Beijing, China) |
| RIPA buffer(high) | R0010 | Solarbio (Beijing, China) |
| Loading buffer | P0015 | Beyotime (Shanghai, China) |
| Running buffer | T1070 | Solarbio (Beijing, China) |
| Tranfer buffer | D1060 | Solarbio (Beijing, China) |
| PVDFmembranes | 3010040001 | Merck (Darmstadt, Germany) |
| PageRuler™ Prestained Protein Ladder | 26616 | Thermo Fisher (Waltham, Massachusetts) |
| Lipofectamine™ 3000 | L3000001 | Thermo Fisher (Waltham, Massachusetts) |
| DMEM-Hams F12 medium | 31765035 | Thermo Fisher (Waltham, Massachusetts) |
| Bovine pituitary extract | Abs9119 | Absin (Wuhan, China) |
| Epidermal growth factor | AF-100-15 | Peprotech(Cranbury, New Jersey) |
| Transferrin | T8158 | Merck (Darmstadt, Germany) |
| Insulin | P3375 | Beyotime (Shanghai, China) |
| Hydrocortisone | HY-N0583 | MCE(New Jersey, US) |
| Penicillin streptomycin | P1400 | Solarbio(Beijing, China) |

**Table S2. List of antibodies**

| **Antibody** | **Catalogue No.** | **Company** | **RRID** |
| --- | --- | --- | --- |
| Rabbit anti-human PTH monoclonal antibody | ab218497 | Abcam (Cambridge, United Kingdom) | AB_3083478 |
| mouse anti-human CD31 monoclonal antibody | #3528 | Cell Signalling Technology (Beverly, MA) | AB_2160882 |
| Rabbit anti-human CD3 monoclonal antibody | #85061 | Cell Signalling Technology (Beverly, MA) | AB_2721019 |
| Rabbit anti-human CD68 monoclonal antibody | ab213363 | Abcam (Cambridge, United Kingdom) | AB_2801637 |
| Rabbit anti-human SMA monoclonal antibody | #19245 | Cell Signalling Technology (Beverly, MA) | AB_2734735 |
| mouse anti-human CASR monoclonal antibody | NB120-19347SS | Novus Biologicals (Centennial, CO) | AB_3083483 |
| Rabbit anti-human CD8 monoclonal antibody | #85336 | Cell Signalling Technology (Beverly, MA) | AB_2800052 |
| mouse anti-human NCAM1 monoclonal antibody | ab6123 | Abcam (Cambridge, United Kingdom) | AB_2149537 |
| Rabbit anti-human EDNRB monoclonal antibody | ab117529 | Abcam (Cambridge, United Kingdom) | AB_10902070 |
| Goat anti-human ACKR1 polyclonal antibody | NB100-2421 | Novus Biologicals (Centennial, CO) | AB_10001541 |
| rabbit anti-human PDGFRA monoclonal antibody | #3174 | Cell Signalling Technology (Beverly, MA) | AB_2162345 |
| mouse anti-human COL6A2 monoclonal antibody | sc-374566 | Santa Cruz Biotechnology (CA, USA) | AB_10991101 |
| mouse anti-human FCER1A monoclonal antibody | NBP1-43278 | Novus Biologicals (Centennial, CO) | AB_3083481 |
| Biotin secondary antibody | SA1020 | BOSTER Biological Technology (Wuhan, Hubei, China) | AB_3083480 |
| goat anti-rabbit IgG-Cy3 | ab97075 | Abcam (Cambridge, United Kingdom) | AB_10679955 |
| goat anti-mouse IgG- Alex 488 | GB25301 | Servicebio (WuHan, Hubei,China) | AB_2904018 |
| Rabbit anti-humanCCND2 monoclonal antibody | ab207604 | Abcam (Cambridge, United Kingdom) | AB_3083479 |
| Rabbit anti-humanKMT2A Polyclonal antibody | #14197 | Cell Signalling Technology (Beverly, MA) | AB_2688010 |
| Rabbit anti-humanGATA3  monoclonal antibody | ab199428 | Abcam (Cambridge, United Kingdom) | AB_2819013 |
| Rabbit anti-human STAT3  monoclonal antibody | ab68153 | Abcam (Cambridge, United Kingdom) | AB_2889877 |
| Rabbit anti-humanβ actin  monoclonal antibody | #4970 | Cell Signalling Technology (Beverly, MA) | AB_2223172 |
| Anti-rabbit IgG, HRP-linked Antibody | #7074 | Cell Signalling Technology (Beverly, MA) | AB_2099233 |

**Table S3. List of siRNAs**

| **Primers** | **Sequences (5'-3')** |
| --- | --- |
| KMT2A siRNA-1 | GAGGCUCACUCUAGAAUAUTT |
| KMT2A siRNA-2 | GGUCCAAACUCCGGAUAAUTT |
| GATA3 siRNA-1 | CCCUGACUAUGAAGAAGGATT |
| GATA3 siRNA-2 | CCCAAGAACAGCUCGUUUATT |
| STAT3 siRNA-1 | GGGACCUGGUGUGAAUUAUTT |
| STAT3 siRNA-2 | CCCGGAAAUUUAACAUUCUTT |

**Table S4. Characteristics of each cell subcluster**

| **Cluster Name** | **Annotated Name** | **Cell Numbers** | **Gene Numbers (mean)** | **UMI Numbers (mean)** | **Mito (mean) %** | **Selected Marker Genes** |
| --- | --- | --- | --- | --- | --- | --- |
| **PC1** | Parathyroid cells | 3619 | 2150.051 | 6203.193 | 8.434384 | *CHGA*  *PTH*  *PTN*  *PVALB*  *RARRES2* |
| **PC2** | Parathyroid cells | 2482 | 3305.242 | 11200.12 | 10.62859 | *RRAD*  *CLDN4*  *ERRFI1*  *ATF3*  *APLP2* |
| **PC3** | Parathyroid adenoma cells | 977 | 3133.696 | 9539.151 | 11.74047 | *SLC31A2*  *TSTD1*  *BCAN*  *RORB*  *MPC2* |
| **PC4** | Parathyroid adenoma cells | 568 | 3639.21 | 12389.49 | 11.52079 | *TTR*  *CD109*  *ATP1B1*  *CYP17A1*  *EI24* |
| **PC5** | Parathyroid cells | 340 | 2823.206 | 8138.318 | 14.09265 | *SLPI*  *CLU*  *KRT19*  *KRT18*  *WFDC2* |
| **PC6** | Parathyroid adenoma cells | 97 | 3190.155 | 10401.64 | 9.928365 | *CASR*  *KL*  *SPOCK3*  *SCPEP1*  *GCM2* |
| **FIB1** | Fibroblasts | 1676 | 1994.288 | 4625.499 | 5.209251 | *DCN*  *APOD*  *CFD*  *LUM*  *SFRP4* |
| **FIB2** | Fibroblasts | 1659 | 1589.219 | 3348.29 | 10.26822 | *RGS5*  *FABP4*  *NDUFA4L2*  *STEAP4*  *IGFBP7* |
| **FIB3** | Fibroblasts | 800 | 2207.281 | 5483.415 | 9.029725 | *TAGLN*  *ACTA2*  *TPM2*  *MYH11*  *MYL9* |
| **FIB4** | Fibroblasts | 284 | 2157.827 | 5049.581 | 4.111201 | *MFAP5*  *IGFBP6*  *FBN1*  *FSTL1*  *PCOLCE2* |
| **EC1** | Capillary endothelial cells | 6232 | 1709.643 | 3437.87 | 4.074262 | *KDR*  *EDNRB*  *FLT1*  *PDGFD*  *PLPP3* |
| **EC2** | Capillary endothelial cells | 5300 | 1717.039 | 3696.348 | 4.345921 | *MT1M*  *CA4*  *SOCS3*  *CAVIN2*  *APOLD1* |
| **EC3** | Venous endothelial cells | 2930 | 1619.538 | 2962.252 | 6.251968 | *ACKR1*  *VWF*  *IGFBP4*  *PLAT*  *SELP* |
| **EC4** | Arterial endothelial cells | 1934 | 1643.705 | 3436.321 | 4.023374 | *IGFBP3*  *CLDN5*  *ENPP2*  *TM4SF1*  *SEMA3G* |
| **EC5** | Venous endothelial cells | 1680 | 2503.053 | 6136.124 | 5.486389 | *SELE*  *C2CD4B*  *ICAM1*  *ADAMTS9*  *AKAP12* |
| **M1** | Macrophage | 2840 | 1549.335 | 4410.869 | 7.48088 | *CCL3L1*  *CCL3*  *CXCL8*  *IL1B*  *RGS1* |
| **M2** | Macrophage | 1758 | 1742.309 | 4236.534 | 7.528134 | *APOE*  *C1QB*  *APOC1*  *C1QC*  *TYROBP* |
| **M3** | Macrophage | 1015 | 1577.075 | 3890.596 | 8.271521 | *MRC1*  *C1QA*  *CD163*  *MS4A7*  *SELENOP* |
| **M4** | conventional DC | 637 | 1760.449 | 4617.429 | 7.278078 | *CST3*  *FCER1A*  *CD1C*  *HLA-DQB1*  *HLA-DRA* |
| **M5** | Monocyte | 497 | 1703.738 | 4208.815 | 6.695312 | *S100A9*  *S100A8*  *LYZ*  *FCN1*  *CTSS* |
| **M6** | monocyte derived DC | 361 | 1683.488 | 4523.704 | 6.875864 | *LST1*  *G0S2*  *BCL2A1*  *AIF1*  *COTL1* |
| **M7** | Macrophage | 119 | 1782.118 | 4518.42 | 6.955524 | *CXCL10*  *IFIT2*  *ISG15*  *MARCKS* |
| **T/NK1** | CD8^+^ T cells | 3650 | 974.2011 | 1979.396 | 6.958067 | *CXCR4*  *IFNG*  *DUSP2*  *UBE2S*  *RUNX3* |
| **T/NK2** | CD16^+^ NK cells and NKT cells | 2171 | 1175.359 | 2298.879 | 6.231864 | *GNLY*  *NKG7*  *GZMB*  *FGFBP2*  *KLRD1* |
| **T/NK3** | CD8^+^ T cells | 1684 | 896.592 | 1487.998 | 6.720701 | *GZMK*  *CCL5*  *CD2*  *IL32*  *TRBC2* |
| **T/NK4** | CD4^+^ T cells | 1416 | 999.2225 | 1966.563 | 5.511837 | *IL7R*  *TNFAIP3*  *RPS29*  *CD48*  *RPS27* |
| **T/NK5** | CD8^+^ T cells | 1259 | 1207.861 | 2445.231 | 5.683804 | *GZMH*  *CD52*  *CD3G*  *CD3D*  *S100A4* |
| **T/NK6** | CD16^-^ NK cells | 567 | 895.0053 | 1679.954 | 8.634073 | *XCL1*  *XCL2*  *AREG*  *KLRB1*  *CD7* |
| **T/NK7** | CD8^+^ T cells | 346 | 1534.225 | 3126.202 | 6.439614 | *TRBC1* |
| **T/NK8** | CD8^+^ T cells | 279 | 1291.91 | 2620.821 | 6.157034 |  |
| **B** | B cells | 642 | 1200.463 | 4249.757 | 6.947053 | *IGKC*  *IGLC2*  *IGHG3*  *IGHA1*  *IGHM* |

**Table S5. List of primers for ChIP and q-PCR**

| **Primers** | **Sequences (5'-3')** |
| --- | --- |
| q-PCR-GATA3-F | GCTCTTGAATTATTTATCGCGTTCC |
| q-PCR-GATA3-R | CCTTGCAGACTTGCCCTCTC |
| q-PCR-KMT2A-F | GGAGAGGATGAGCAATTCTTAG |
| q-PCR-KMT2A-R | GGAGACCTTGTGGGACTT |
| q-PCR-GATA3-F | GTCCTGTGCGAACTGTCAGA |
| q-PCR-GATA3-R | TCGGTTTCTGGTCTGGATGC |
| q-PCR-CCND2-F | TTAAGAACCGGAGGCAAAG |
| q-PCR-CCND2-R | CCTTCCAGAGGAGAGAGTT |
| q-PCR-STAT3-F | ACCAACGACCTGCAGCAATA |
| q-PCR-STAT3-R | GGACTCAAACTGCCCTCCTG |
| q-PCR-RPL13A-F | CCTGGAGGAGAAGAGGAAAGAGA |
| q-PCR- RPL13A -R | TTGAGGACCTCTGTGTATTTGTCAA |


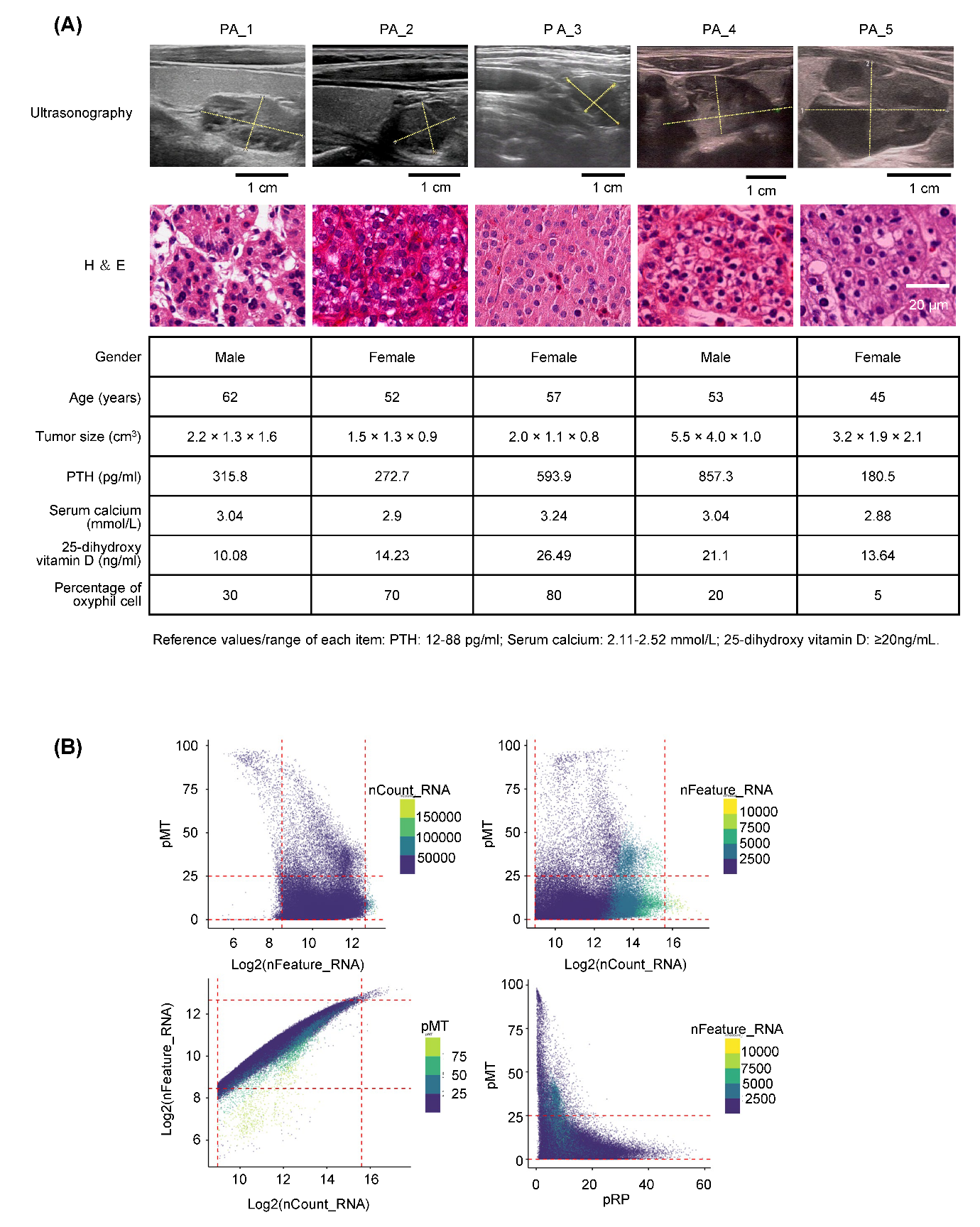
**Figure S1. Clinical and pathological information of PA cases and quality control of scRNA-seq data.** (A) Representative ultrasonographic images of PAs and the microphotographs of H&E staining of the corresponding FFPE tissue sections verifying clinical and pathological diagnosis of the PA cases included in this study. Relevant cinicopahological information is also shown. (B) Scatter plots of quality control parameters of all single-cell transcriptomes analyzed in the study, with red dashed lines indicating cut-off values for filtering of high-quality transcripts.


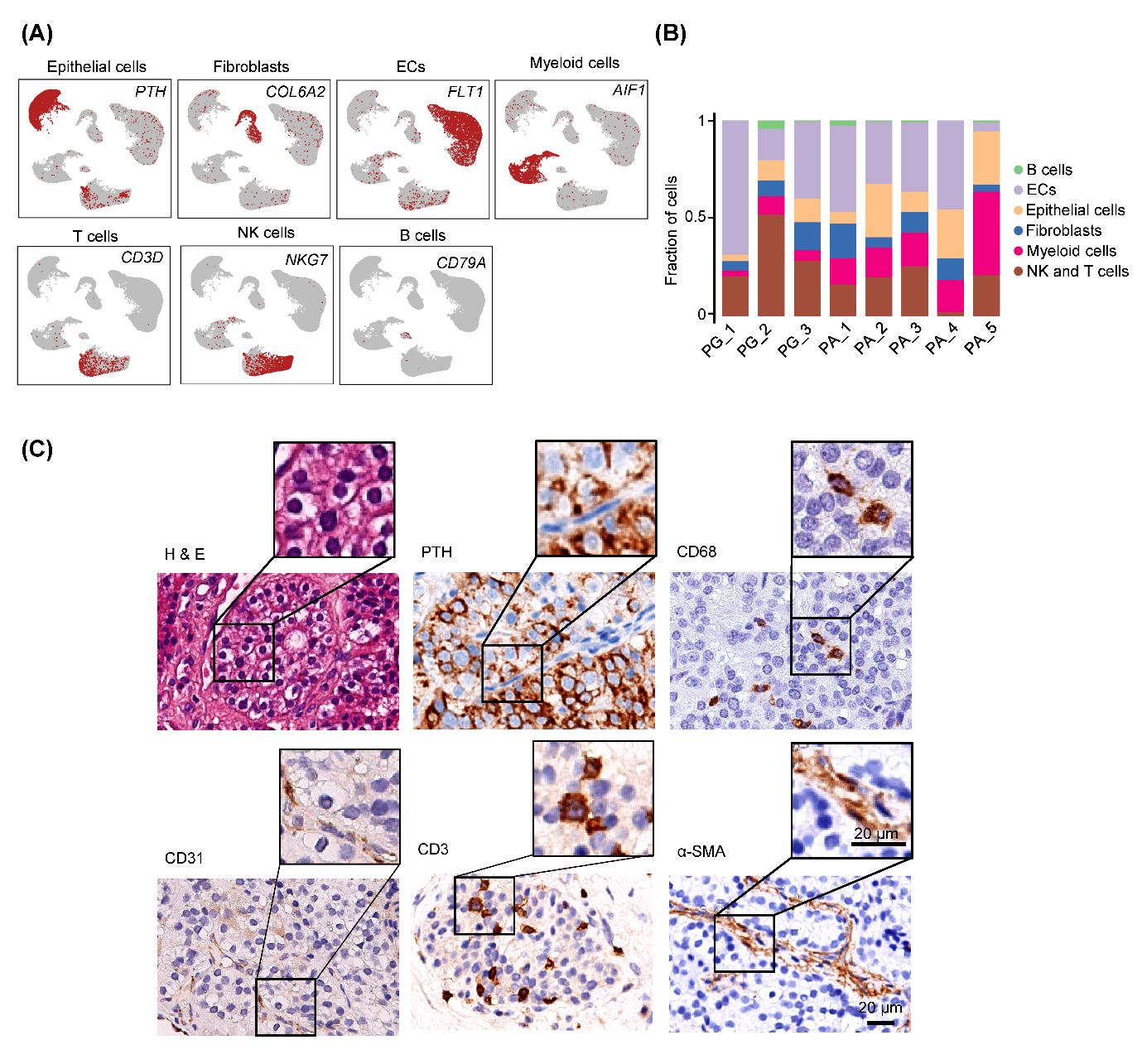
**Figure S2**. **Cell typing of PA relative to PG tissues through scRNA-seq**. **(A)** UMAP plots pseudocolored for the expression of the marker genes of the major cell types. **(B)** Proportion plots of major cell types in PA (PA_1-5) and PG (PG_1-3) tissues derived from different individuals. **(C)** Representative microphotographs of H&E staining and IHC staining of PTH (PACs), CD68 (myeloid cells), CD31 (endothelial cells), CD3 (T cells), and α-SMA (fibroblasts) on FFPE PA tissue sections from independent patients with sporadic PAs (n = 12).


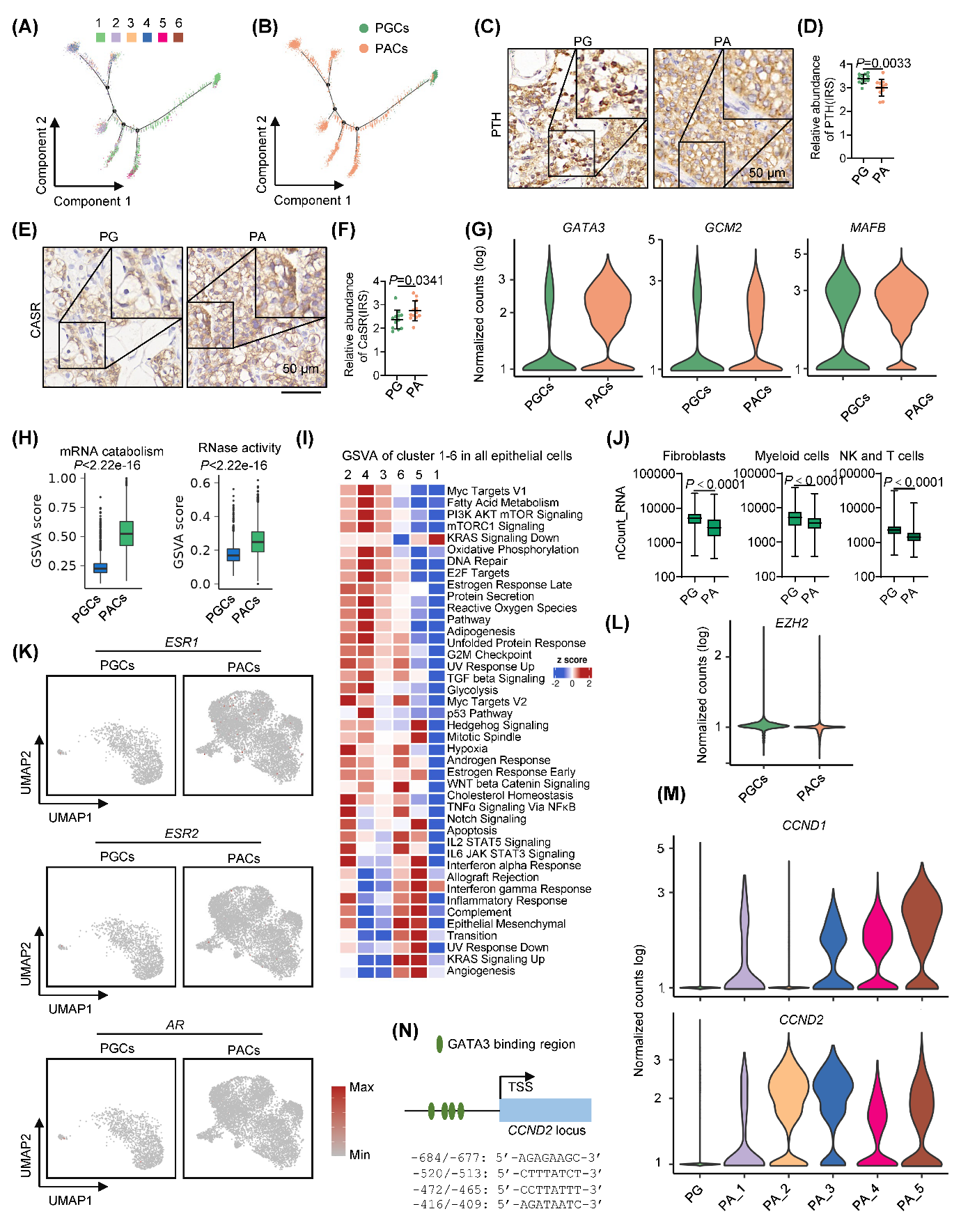


**Figure S3**. **Characteristics of PACs versus PGCs**. **(A & B)** Development trajectory of epithelial cells inferred by diffusion map, colored by clusters (A) and tissue origin (B). **(C)** Representative microphotographs of IHC staining of PTH on FFPE PA (n = 12) and PG (n = 12) tissue sections. **(D)** Quantitation of PTH expression in PA (n = 12) and PG (n = 12) tissues as shown in C. Data shown are mean immunoreactive score (IRS) ± s.d. Two-tailed Student’s *t*-test. **(E)** Representative microphotographs of IHC staining of CASR on FFPE PA (n = 12) and PG (n = 12) tissue sections. **(F)** Quantitation of PTH expression in PA (n = 12) and PG (n = 12) tissues as shown in E. Data shown are mean immunoreactive score (IRS) ± s.d. Two-tailed Student’s *t*-test. **(G)** Violin plots of the expression distribution of *GATA3, GCM2* and *MAFB* in PACs and PGCs (n = 6,589 and 1,494 cells for PACs and PGCs, respectively). **(H)** Boxplots showing the differential GSVA scores summarizing the expression of genes involved in mRNA catabolic processes (GO:0061014) and in ribonuclease activity (GO:0004540), in PACs and PGCs. The plot centres, box and whiskers correspond to median, interquartile range (IQR) and 1.5 × IQR, respectively (n = 6,589 and 1,494 cells for PACs and PGCs, respectively). **(I)** Heatmap of the differential expression of hallmark pathway signature genes scored per cell using GSVA among different epithelial cell clusters. Data shown are z scores from a liner model. **(J)** Box plots of the number of transcripts detected in fibroblast, myeloid cells and NK and T cells of PA and PG microenvironments (with plot center, box and whiskers corresponding to median, IQR and 1.5 × IQR, respectively; n = 2,878 and 1,541 cells for PA and PG fibroblasts; n = 6,156 and 1,071 cells for PA and PG myeloid cells; n = 5,381 and 5,986 cells for PA and PG T and NK cells, respectively). **(K)** UMAPs of PACs and PGCs, color-coded for the expression of *AR*, *ESR1*, and *ESR2*. **(L)** Violin plots of the expression distribution of *EZH2* in epithelial cells of PACs and PGCs (n = 6,589 and 1,494 cells for PACs and PGCs, respectively). **(M)** Violin plots of the expression distribution of *CCND1* and *CCND2* in PACs from individual patients and PGCs shown in total of those from 3 individuals (n = 274, 2,892, 579, 1,681, and 1,163 cells for PACs from PA_1, PA_2, PA_3, PA_4, and PA_5, and 1,494 cells for PGCs, respectively). **(N)** Schematic illustration of the four consensus GATA3 binding regions (green oval) located to the-684/677, -520/513, -472/465 and -416/409 regions of the proximal promoter of *CCND2* gene.


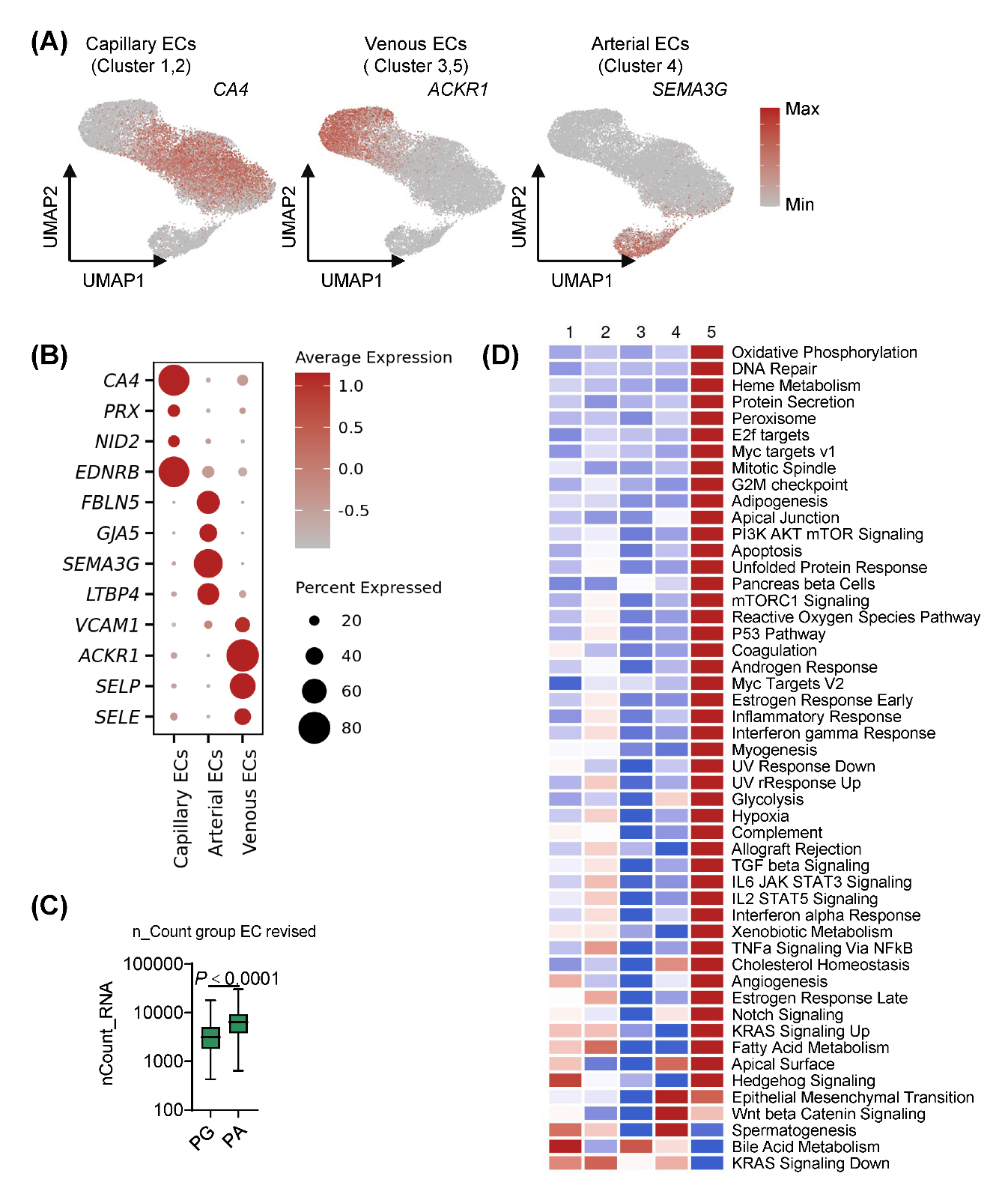
**Figure S4**. **Characteristics of ECs in the PA microenvironment. (A)** UMAP plots of the 8 EC clusters with each cell color-coded for the expression of a marker gene for the individual cell subtypes (*CA4* for capillary ECs, *ACKR1*, venous Ecs, and *SEMA3G* for arterial ECs). **(B)** Dot plots of the expression of marker genes of capillary, arterial and venous ECs. Expression values are normalized to the scaled averages. **(C)** Box plots of the number of transcripts detected in ECs of PA and PG microenvironments (with plot center, box and whiskers corresponding to median, IQR and 1.5 × IQR, respectively; n = 10,724 and 7,352 ECs, respectively). **(D)** Heatmap of the differential expression of hallmark pathway signature genes scored per cell using GSVA among different EC clusters. Data shown are z scores from a liner model.


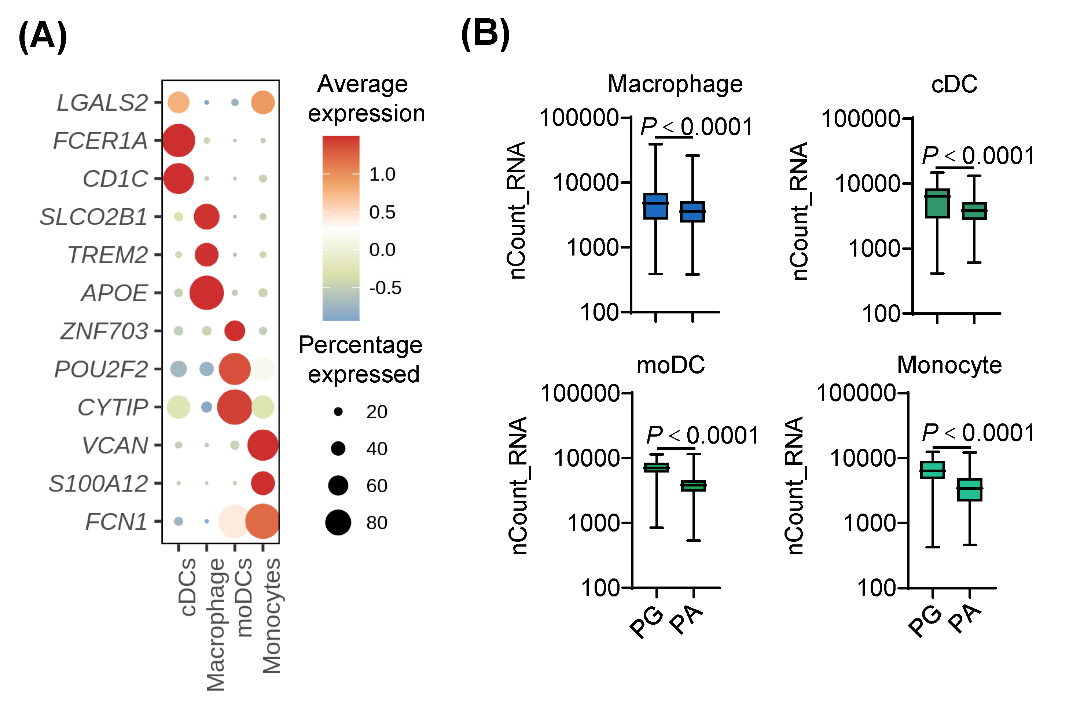


**Figure S5**. **Characteristics of myeloid cells in the PA microenvironment. (A)** Dot plots of the expression of marker genes of cDCs, macrophages, moDCs, and monocytes. Expression values are normalized to the scaled averages. **(B)** Box plots of the number of transcripts detected in macrophages, cDCs, moDCs, and monocytes from PA and PG microenvironments (with plot center, box and whiskers corresponding to median, IQR and 1.5 × IQR, respectively.).


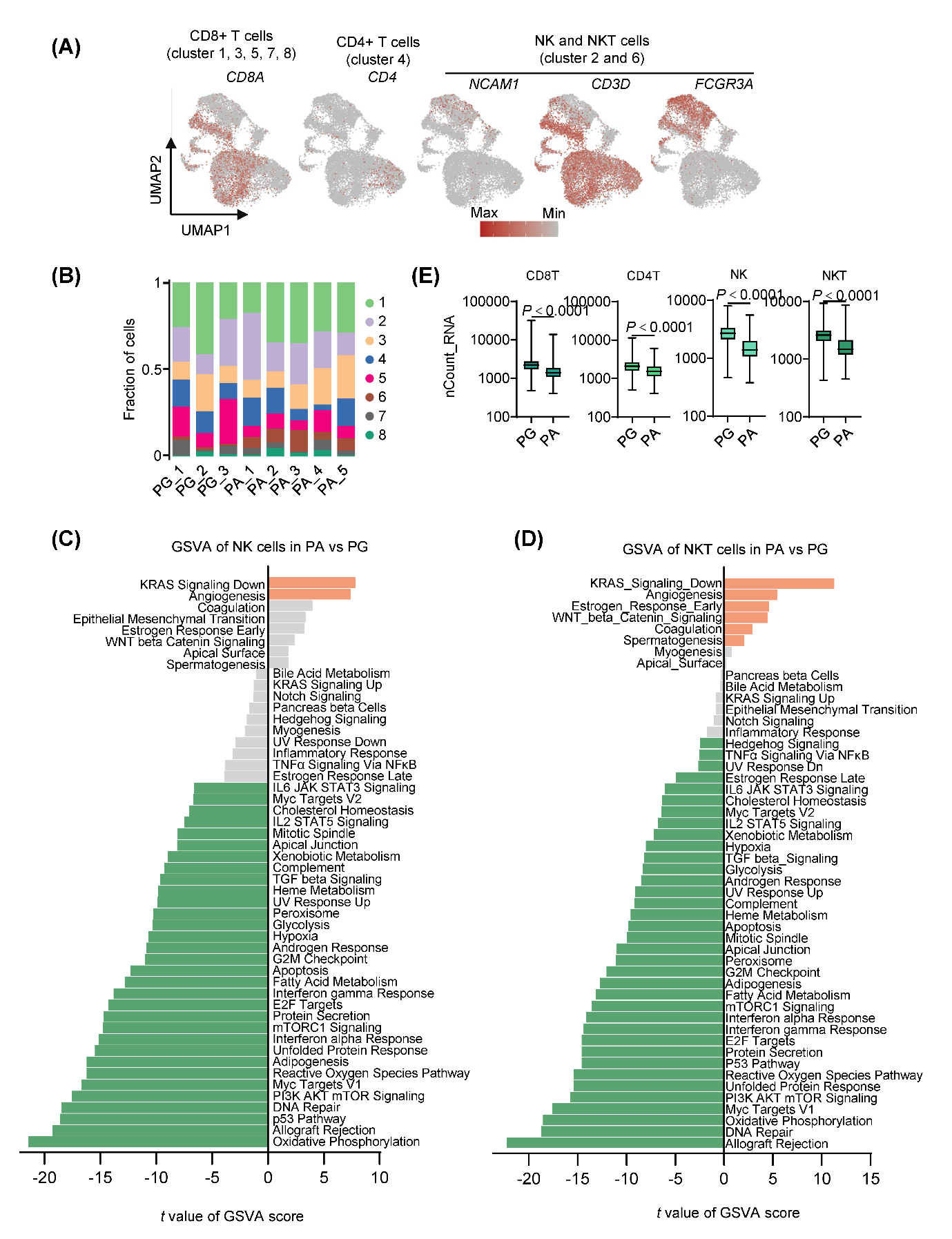


**Figure S6. Characteristics of T and NK cells in the PA microenvironment. (A)** UMAP plots of the 8 NK and T cells clusters with each cell color-coded for the expression of a marker gene for the individual cell subtypes (*CD8A* for CD8+ T cells, *CD4* for CD4+ T cells, *NCAM1* for NK, and *NCAM1* as well as *CD3D* for NKT cells). **(B)** Proportion plots of the 8 clusters of T and NK cell recovered from PA (PA_1, PA_2, PA_3, PA_4, and PA_5) and PG (PG_1, PG_2, and PG_3) microenvironments. **(C & D)** Differential expression of hallmark pathway gene signatures scored per cell using GSVA in PA versus PG NK cells (C; n = 1,137 and 1,601 cells for PA and PG NK cells, respectively) and NKT cells (D; n = 1,196 and 688 cells for PA and PG NKT cells, respectively ). Data shown are *t* values from a linear model. Dn, down; UV, ultraviolet; v1, version 1; v2, version 2. **(E)** Box plots of the number of transcripts detected in CD8 T, CD4 T, NK and NKT cells from PA and PG microenvironments (with plot center, box and whiskers corresponding to median, IQR and 1.5 × IQR).


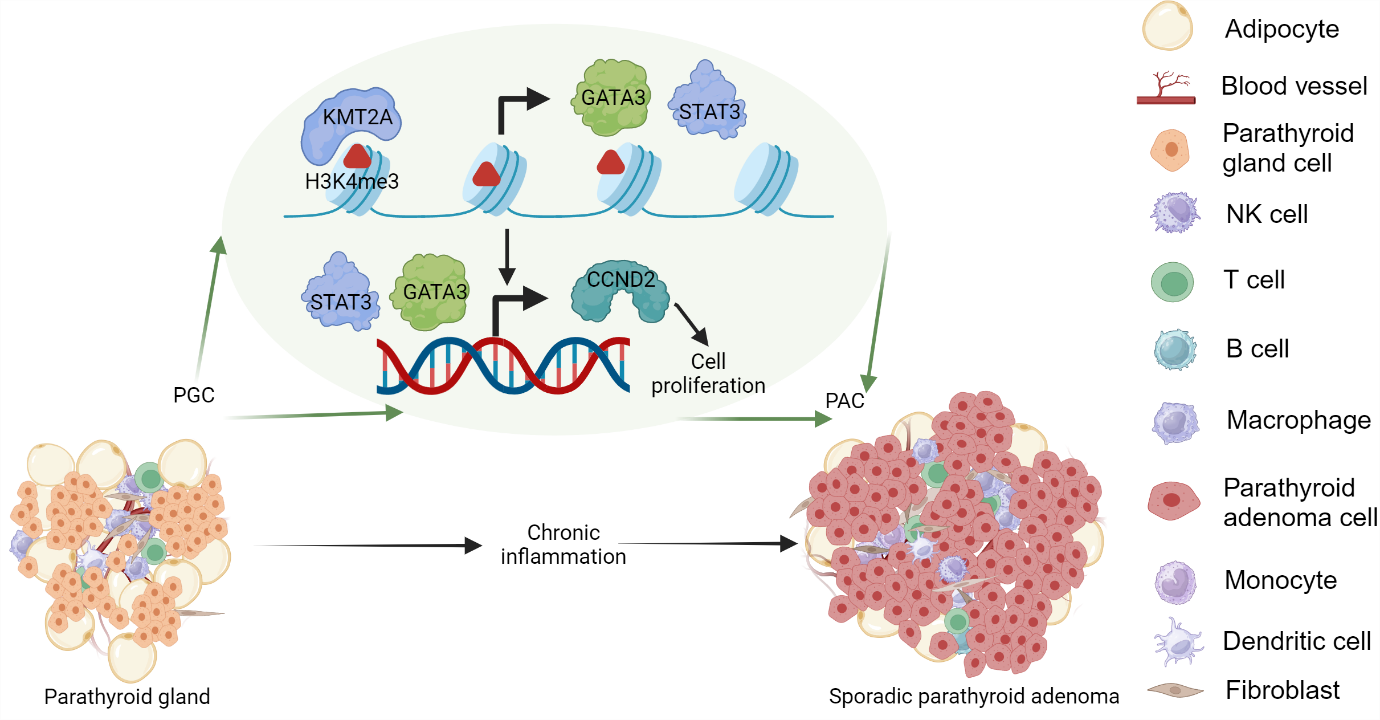


**Figure S7.** Schematic illustration of a hypothetical model for the pathogenesis of PAs: KMT2A-mediated epigenetic mechanism drives a pervasive transcriptional increase in gene expression, leading to the upregulation of STAT3 and GATA3, which in turn transactivate CCND2 to promote PACs proliferation, whereas a chronic inflammatory microenvironment promotes PA pathogenesis through mechanisms such as angiogenesis and immune suppression.
